# Exposures to humidifier disinfectant and various health conditions in Korean based on personal exposure assessment data of claimants for compensation

**DOI:** 10.1186/s12889-023-16389-x

**Published:** 2023-10-02

**Authors:** Myeongjin Hong, Min Jae Ju, Jeonggyo Yoon, Wonyoung Lee, Seula Lee, Eun-kyung Jo, Seo-Youn Choi, Wonho Yang, Yoon-Hyeong Choi

**Affiliations:** 1 Department of Preventive Medicine, Gachon University College of Medicine, Incheon, Korea; 2 School of Health and Environmental Science, College of Health Science, Korea University, 145 Anam-Ro, Seongbuk-Gu, 02841 Seoul, Republic of Korea; 3 Department of Community, Environment and Policy, Mel and Enid Zuckerman College of Public Health, University of Arizona, Tucson, AZ USA; 4 Korean Society of Environmental Health, Seoul, Korea; 5 Department of Radiology and Center for Imaging Science, Samsung Medical Center, Sungkyunkwan University School of Medicine, Seoul, Korea; 6 Department of Occupational Health, Daegu Catholic University, Gyeongbuk, Korea

**Keywords:** Humidifier disinfectant, Humidifier disinfectant exposure, Reported health conditions

## Abstract

**Background:**

Humidifier disinfectants (HDs) were commonly used household chemicals to prevent microbial growth in a humidifier water tank in South Korea. A growing body of evidence has indicated that its airborne exposure can induce severe lung injury. However, there has been low awareness of other health outcomes in HD users. This study aimed to evaluate health conditions appealed by claimants for compensation in relation with an increased exposure to HD.

**Methods:**

From survey data of personal HD exposure assessment of claimants for compensation in Korea, we included a total of 4,179 subjects [cases in each dataset were defined by nine reported health conditions, i.e., pneumonia, asthma, cardiovascular disease, respiratory disease, otorhinolaryngologic disease, brain disease (including cerebrovascular disease), dermatological disease, lung cancer, and all cancers]. HD exposures was considered as the following exposure criteria: exposure duration, exposure proximity, exposure direction, chemical type, cumulative exposure time, indoor air concentration, and cumulative exposure level. Logistic regression models were used to evaluate the associations between HD exposure and health conditions.

**Results:**

After adjusting for sociodemographic and health behavioral factors and other chemical exposures (households, environmental, and occupational exposures), an increase in cumulative HD exposure time was significantly associated with risks of all nine diseases (all *p*-trends < 0.05). An increase in HD exposure duration was associated with asthma, respiratory disease, otorhinolaryngologic disease, dermatological disease, all cancers, and lung cancer (*p*-trends < 0.05). Indoor HD concentration was associated with only pneumonia (*p*-trend = 0.015).

**Conclusions:**

Our findings suggest that cumulative exposures to airborne HD might potentially increase the risk of various reported health outcomes.

**Supplementary Information:**

The online version contains supplementary material available at 10.1186/s12889-023-16389-x.

## Introduction

Humidifier disinfectants (HDs) were first invented in South Korea in 1994 to prevent microbial growth as a household chemical additive to water in a humidifier [1]. HDs added to water are released into the air as chemical-containing water vapor, which can cause airborne exposure to people via inhalation. The main chemical components of HDs are polyhexamethylene guanidine (PHMG, CAS No. 31961-54-3), oligo-(2-(2-ethoxy)-ethoxyethyl) guanidine (PGH, CAS No. 374,572–91-5), or a mixture of chloromethylisothiazolinone (CMIT, CAS No. 26,172–55-5) and methylisothiazolinone (MIT, CAS No. 2682–20-4), although chemicals might differ depending on the products [2]. Case series of unknown fatal lung injuries were reported in children in 2006 [3] and adults in 2011 [4]. In 2011, the Korean Centers for Disease Control and Prevention [1] conducted epidemiological investigation of those cases and suggested that their lung injuries, including interstitial pneumonitis and widespread lung fibrosis, were associated with HD use [5]. Finally, the Korean government banned sales of HDs in the market and commanded companies to recall their products. Until their sales were banned, more than 6 million people were estimated to have used HD products [6].

To date, a huge number of humidifier disinfectant-associated lung injuries (HDLIs) and potential adverse health outcomes, i.e., a total of 7,830 claimants for compensation (as of March 31st, 2023), have been reported to determine whether their health conditions might be associated with HD use in Korea [7]. There have been growing in vivo and in vitro investigations to evaluate whether each of HD chemical components affects lung injuries [8–10]. However, there is low awareness of other health effects in HD users, although previous experimental studies have indicated biological plausibility that: 1) exposure of PHMG causes apoptotic cell death and inflammatory response in human liver epithelium cells [11], 2) exposures to PHMG and PGH can cause severe atherosclerotic changes with a cytotoxic effect on human dermal cells and induce severe vascular fibrosis, inflammation, and embryonic toxicity [12], 3) exposure to CMIT/MIT can induce T helper 2-related dysfunction and enhance atopic dermatitis-like phenotypes in mice [13], and 4) exposure to MIT can induce apoptotic cell death along with membrane damage in human liver epithelial cells [14]. Recently, only a few epidemiologic studies using Korean children panels have suggested that HD exposures might be associated with bronchiolitis [15], allergic rhinitis [16], and asthma [17] in children aged 1–7 years. The findings of these studies primarily focused on the respiratory diseases; however, there is a necessity for further epidemiological research to explore possible associations of HD with various diseases on other organs.

Therefore, the present study aimed to assess whether HD-exposed people including children-to-adolescents and adults might have experienced the reported health conditions (i.e., pneumonia, asthma, cardiovascular disease, respiratory disease, otorhinolaryngologic disease, brain disease, dermatological disease, lung cancer, and all cancers) other than lung injuries, since they had been exposed to HD, using personal exposure assessment data for HD-exposed population in Korea. This study also examined the dose-dependent associations between HD exposure and the prevalence of each reported health condition while controlling for important confounding factors including sociodemographic and health behavioral factors as well as other households, environmental, and occupational exposures.

## Methods

### Study population

**Fig. 1:**
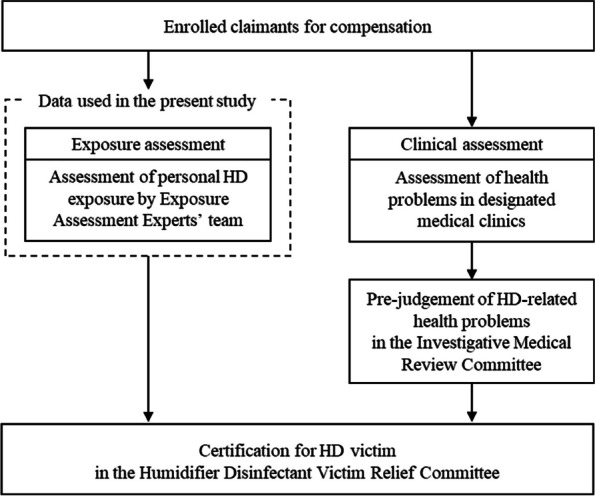
Process of victim certification on claimants for compensation

**Fig. 2:**
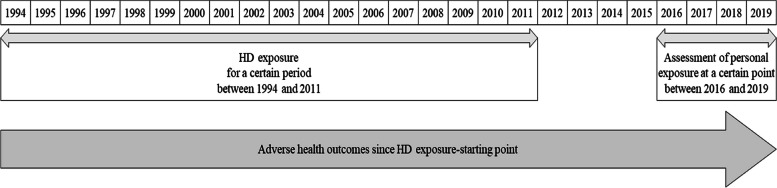
Timeline between exposure and assessment

This study used data from HD exposure assessment on claimants for compensation to the government conducted by the Korea Environmental Industry and Technology Institute of the Korea Ministry of Environment [7]. Data were purposed to be used to certificate HD claimants in accordance with the act, “Special Act on Remedy for Damage Caused by Humidifier Disinfectants”, in which their exposure and clinical histories have been reviewed by the committee for the determination of HD-related health effects (For a brief process, see Fig. 1) [7]. Data were collected through extensive standardized interviews by occupational and environmental health nurses or environmental hygienists trained and certified by the Korean Society of Environmental Health, in order to obtain HD exposure history (a certain period between 1994 and 2011) and medical history (since HD exposure-starting point; see Fig. 2). In addition, sociodemographic and physical information and household environment and occupational exposure information were obtained [18]. All claimants in the HD exposure assessment provided written informed consent (or their parent in the case of children and adolescents under 19), consistent with approval by Daegu Catholic University Institutional Review Board (IRB No. CUIRB-2016–0114).

**Fig. 3:**
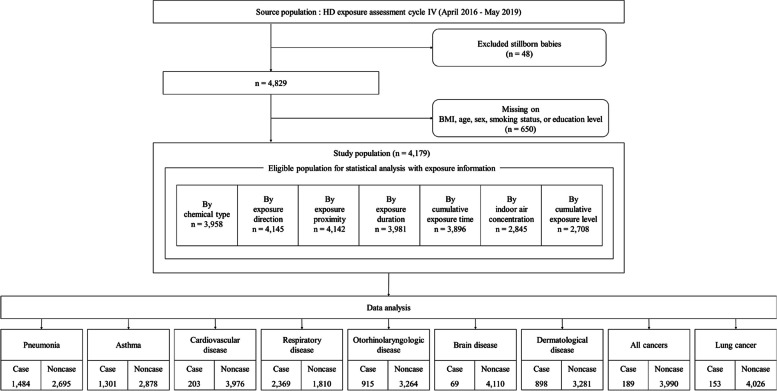
Flow diagram showing the selection of the study population

In HD exposure assessment, because information for reported health conditions has been available since April 2016 (cycle IV), the present study included an initial sample (namely, source population) of 4,877 subjects enrolled for the HD exposure assessment between April 2016 and May 2019 (cycle IV-i, ii, iii). We excluded 48 claimants of stillborn babies for whom information for health conditions could not be obtained for this study. Subjects with missing information on covariates (*n* = 650) were also excluded from the statistical analysis, resulting in a total of 4,179 subjects (subjects lacking data on each of exposure information were excluded from statistical analysis (see Fig. 3).

This study included nine kinds of analyses using personal HD exposure assessment data; cases in each set were defined by nine reported health conditions, i.e., pneumonia (1,484 cases and 2,695 non-cases), asthma (1,301 cases and 2,878 non-cases), cardiovascular disease (203 cases and 3,976 non-cases), respiratory disease (2,369 cases and 1,810 non-cases), otorhinolaryngologic disease (915 cases and 3,264 non-cases), brain disease [including cerebrovascular disease (69 cases and 4,110 non-cases)], dermatological disease (898 cases and 3,281 non-cases), all cancers (189 cases and 3,990 non-cases), and lung cancer (153 cases and 4,026 non-cases, Fig. 3).

### Exposures to humidifier disinfectants

HD exposures of each claimant were accessed with standardized extensive interviews including a variety of HD exposure-related questionnaires. Claimants were asked about their exposure histories between 1994 and 2011. Questionnaires for personal HD exposure assessment consisted of HD product name, use amount, frequency and a bundle of usage behaviors in separate survey sheets organized by each person, period, and space (room in housing or office). In the current study, we considered seven exposure criteria (four single criteria and three computed criteria): 1) chemical type, chemical component of HD product which each participant normally used, was categorized into PHMG, PGH, CMIT/MIT, and others (each participant was asked to report HD product’ name, and we assigned its chemical component based on prior literature) [19]; 2) exposure direction (i.e., humidifier spray direction) was categorized as toward the face or not; 3) exposure proximity defined as the distance from humidifier to subject’s face was categorized into ≥ 1, 0.5 to < 1, or < 0.5 m; 4) exposure duration (month) was categorized into < 6, 6 to < 12, 12 to < 24, or ≥ 24 months; 5) cumulative exposure time (hour) was computed by multiplying the years of use (year), annual use (months/year), monthly use (weeks/month), daily use (days/week), and hourly use (hours/day); 6) indoor air concentration (μg/m^3^) (i.e., indoor HD concentration) was computed by multiplying the used HD product concentration by the daily average usage amount and dividing by the volume of the space where used, using a method suggested by Park et al [20]. Claimants with missing information on HD brand, usage amount, and space were excluded from calculations. Claimants who used HD products without available information on chemical concentrations were additionally excluded from calculations; 7) cumulative exposure level (μg/m^3^·hr) was computed by multiplying cumulative exposure time and indoor air concentration. The last three criteria were categorized into as quartiles [20].

### Reported health conditions

Health conditions were defined by self-reported physician’s diagnosis for each participant. Claimants were asked to report “Choose any health condition(s) which has been developed after HD uses from options below and describe the details.”. They answered the development of one or more health conditions. Current study used nine diseases of pneumonia, asthma, cardiovascular disease, respiratory disease, otorhinolaryngologic disease, brain disease, dermatological disease, lung cancer, and all cancers (see Supplementary Table 1).

### Covariates

Age, body mas index (BMI), sex, smoking status, and education level were considered as potential confounders, which needed to be controlled for, based on biological consideration for which nine diseases share similarities. Age was defined as age of claimants at which they experienced health effects after HD exposure (continuous). BMI was calculated by dividing the weight in kilograms by the height in meters squared (continuous). Smoking status was categorized as never smoker (reference), former smoker, or current smoker. Education level was categorized as less than high school (reference), high school graduate, and college graduate or higher.

### Other environmental factors

Other environmental and occupational exposures during the one year prior to their adverse health effects were also considered as potential confounders. Exposure to commercial chemicals was computed by the number of chemicals that claimants used among the following 16 household chemicals: fabric brightener, household insecticides, deodorant or air freshener, water repellent, disinfectant or sanitizer, paint additive, glass cleaner, mothball, glue, stain remover, dishwashing detergent, laundry detergent, scented candle or fumigant, hair styling product, at-home dry cleaner, and polish (score of 0 to 16). Exposures to house mold and neighboring factories or incinerator were defined as exposed by questionnaire if the subject had ever had damp stains or molds on the walls or ceiling of the house and if the subject had ever resided within 1 km of facilities such as factories or incinerators. Occupational exposure was defined as exposed by a questionnaire if the subject had ever been exposed to toxic gas, fumes, or smoke in the workplace.

### Statistical analysis

All statistical analyses were performed using SAS software version 9.4 (SAS Institute Inc., Cary, NC, USA). A value of *p* < 0.05 was deemed to be statistically significant. Logistic regressions models were used to estimate the odds ratios (ORs) for the risks of nine diseases with each HD exposure in separate models. All seven independent variables of HD exposures were modeled as categorical variables. Chemical type was modeled with PHMG which had the largest number of exposed claimants as a reference, while other six exposure variables were modeled with the lowest exposed group as a reference. We also computed *p*-values for linear trend by fitting the exposure as an ordinal variable coded using integer values (0–3 or 0–2). All models were adjusted for BMI, age, sex, smoking status, and education level as covariates. Furthermore, in order to show variation of the estimated ORs between seven exposures and nine diseases, a graphical model was developed with a heatmap using R software version 4.2.1 (“ggplot2”, “ggpubr” packages).

### Sensitivity analysis

Sensitivity analysis was conducted with additional adjustment for environmental and occupational exposures affecting health conditions as potential confounders. Thus, each of commercial chemical, house mold, neighboring factory or incinerator, and occupational exposure was further controlled in separated models and each other in one model, i.e., multi-pollutant model.

Another sensitivity analysis was conducted with age-stratified models, and their details are available in the Supplementary Material, “Age-stratified Analysis: Methods”.

## Results

Table 1 shows general characteristics of study population (*n* = 4,179). Overall, mean ± standard error (SE) of age was 33.1 ± 24.9 years. Males were 52.0 % (*n* = 2,172), and current survivors were 83.6 % (*n* = 3,495). By chemical type of HD products, most claimants used PHMG-containing HD (76.3 %, *n* = 3,021), followed by those who used CMIT/MIT (19.6 %, *n* = 775) and those who used PGH (1.1 %, *n* = 44). Claimants had various diseases developed after HD uses, including pneumonia (35.5 %, *n* = 1,484), asthma (31.1 %, *n* = 1,301), cardiovascular disease (4.9 %, *n* = 203), respiratory disease (56.7 %, *n* = 2,369), otorhinolaryngologic disease (21.9 %, *n* = 915), brain disease (1.7 %, *n* = 69), dermatologic disease (21.5 %, *n* = 898), all cancers (4.5 %, *n* = 189), and lung cancer (3.7 %, *n* = 153). General characteristics between subjects with and without each of the nine reported health conditions were compared in Supplementary Material Tables S2 - S10. Supplementary Material Table S11 shows exposure characteristics, including chemical type, exposure direction, exposure proximity, exposure duration, indoor air concentration, cumulative exposure time, and cumulative exposure level in subjects with each nine of reported health conditions.

**Table 1:** Characteristics of claimants (*n* = 4,179)

Characteristics	Distribution	
Age [years]	33.1	± 24.9
BMI [kg/m^2^]	21.7	± 4.1
Sex		
Male	2,172	(52.0)
Female	2,007	(48.0)
Survival status		
Survivors	3,495	(83.6)
Non-survivors	684	(16.4)
Cigarette smoking		
Never smoker	3,164	(75.7)
Former smoker	856	(20.5)
Current smoker	159	(3.8)
Education		
< High school	1,869	(44.7)
High school	1,020	(24.4)
> High school	1,290	(30.9)
Chemical type^a^		
PHMG	3,021	(76.3)
PGH	44	(1.1)
CMIT/MIT	775	(19.6)
Others	118	(3.0)
Reported health conditions^b^		
Pneumonia	1,484	(35.5)
Asthma	1,301	(31.1)
Cardiovascular disease	203	(4.9)
Respiratory disease	2,369	(56.7)
Otorhinolaryngologic disease	915	(21.9)
Brain disease	69	(1.7)
Dermatological disease	898	(21.5)
All cancers	189	(4.5)
Lung cancer	153	(3.7)
Environmental exposures		
Number of chemical usage^c^	4.0	± 2.3
House mold	255	(6.1)
Neighborhood factory or incineration	87	(2.1)
Occupational exposure	59	(1.4)

Data in tables are presented as mean ± SE for continuous variables and sample size (percentage) for categorical variables. Brain disease: brain and cerebrovascular disease

^a^Subsample of participants with available information in chemical type of humidifier disinfectants (*n* = 3,958)

^b^Comorbid health conditions were defined as self-reported diagnosis of each disease

^c^Number of chemicals usage: fabric brightener, household insecticides, deodorant or air freshener, water repellent, disinfectant or sanitizer, paint additive, glass cleaner, mothball, glue, stain remover, dish washing detergent, laundry detergent, scented candle or fumigant, hair styling product, at-home dry cleaner, and polish (range 0–16)

Abbreviations: BMI, body mass index; PHMG, polyhexamethylene guanidine; PGH, oligo (2- (2-) ethoxy ethoxyethyl) guaindinium; CMIT, chloromethylisothiazolionoe; MIT, methylisothiazolinone

**Fig. 4:**
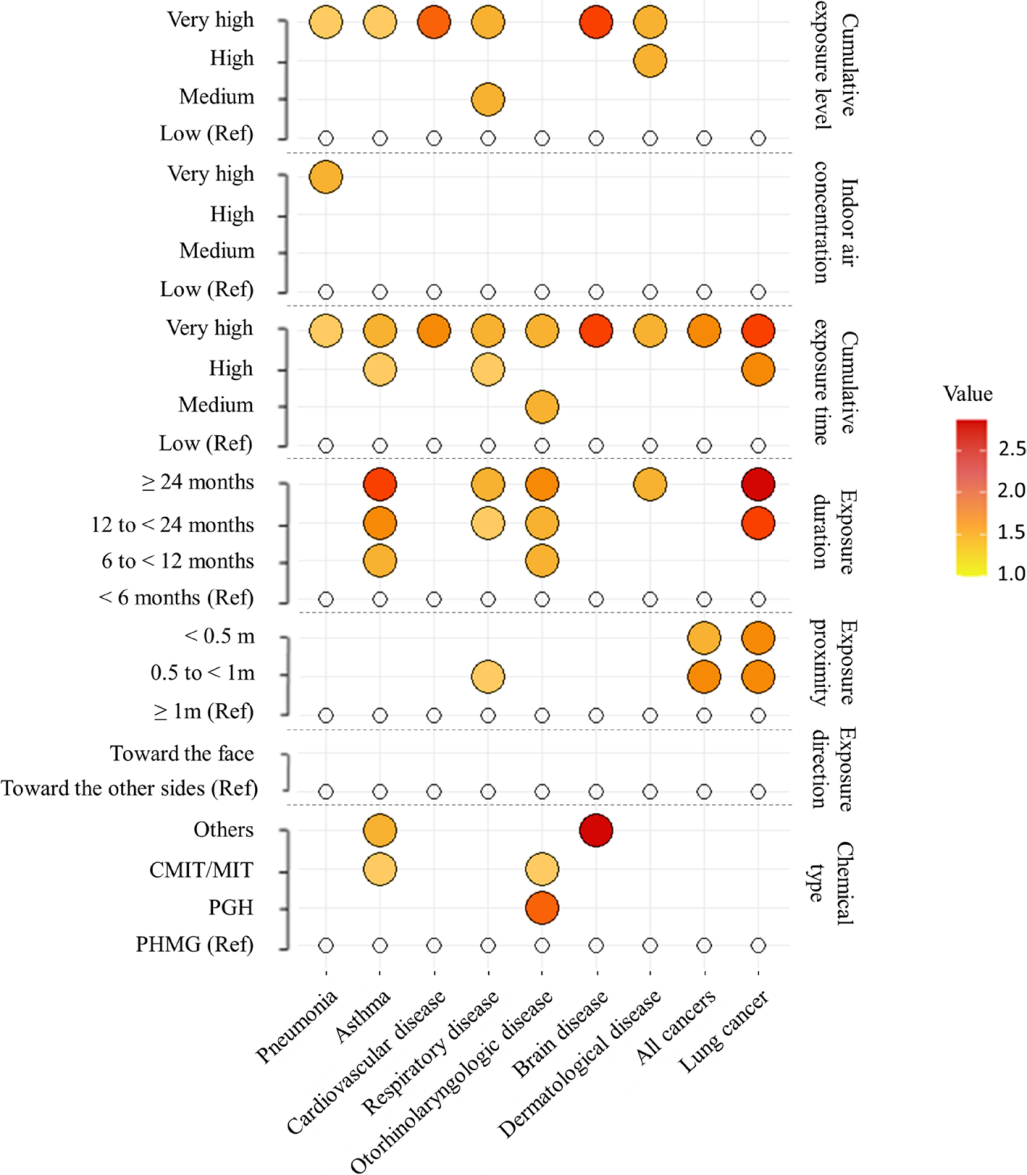
Heatmap of a OR matrix of reported health conditions by HD exposures. All values were from Table 3, and only significant ORs were presented in the heatmap. The redder colors indicate the higher ORs

Table 2 presents results of logistic regression models for reported health conditions of subjects with HD exposures after adjusting for sociodemographic and health behavioral factors. All nine diseases were dose-dependently associated with cumulative exposure time (all *p-*trends < 0.05). Fully adjusted OR comparing the highest quartile versus the lowest quartile of cumulative exposure time was 1.25 (95% CI: 1.03–1.52) for pneumonia, 1.40 (95% CI: 1.14–1.71) for asthma, 1.83 (95% CI: 1.21–2.76) for cardiovascular disease, 1.51 (95% CI: 1.26–1.82) for respiratory disease, 1.44 (95% CI: 1.14–1.82) for otorhinolaryngologic disease, 2.17 (95% CI: 1.05–4.46) for brain disease, 1.54 (95% CI: 1.23–1.93) for dermatological disease, 1.63 (95% CI: 1.04–2.56) for all cancers, and 2.03 (95% CI: 1.21–3.42) for lung cancer. Additionally, asthma and dermatological diseases showed significant dose-dependent associations with exposure duration and cumulative exposure level (all *p-*trends < 0.05). Pneumonia showed significant dose-dependent associations with indoor air concentration and cumulative exposure level (*p-*trend < 0.05). Cardiovascular disease showed a significant dose-dependent association with cumulative exposure level (*p-*trend = 0.014). Respiratory disease showed significant dose-dependent associations with exposure duration and cumulative exposure level (*p-*trend < 0.001 and = 0.001, respectively) and a significant association with shorter exposure proximity (with 0.50 to < 1 m vs. ≥ 1 m). Cancer (all cancers) and lung cancer showed significant dose-dependent associations with exposure duration (*p*-trend: = 0.019 and = 0.001, respectively) and significant associations with shorter exposure proximity (with < 1 m vs. ≥ 1 m). Otorhinolaryngologic disease showed significant dose-dependent associations with exposure duration and indoor air concentration (*p-*trend < 0.001) and a significant association with HD chemical type (with PGH and CMIT/MIT vs. PHMG). Brain disease showed significant dose-dependent associations with cumulative exposure level (*p-*trend = 0.014) and a significant association with HD chemical type (with others vs. PHMG). In order to better understand results mentioned above, all their ORs were graphically presented in Fig. 4. Furthermore, their associations in subgroups stratified by age (i.e., children-to-adolescents and adults) are available in the Supplementary Material, “Age-stratified Analysis: Results” (Supplementary Material, Figure S1).

**Table 2:** ORs (95% CIs) of reported health conditions by humidifier disinfectant exposures

Exposures	Pneumonia		Asthma		Cardiovascular disease		Respiratory disease		Otorhinolaryng-ologic disease		Brain disease		Dermatological disease		All cancers		Lung cancer	
Chemical type (*n* = 3,958)																		
PHMG	1.00	(Reference)	1.00	(Reference)	1.00	(Reference)	1.00	(Reference)	1.00	(Reference)	1.00	(Reference)	1.00	(Reference)	1.00	(Reference)	1.00	(Reference)
PGH	0.79	(0.41–1.51)	0.87	(0.45–1.68)	1.23	(0.28–4.92)	0.76	(0.42–1.39)	2.00	(1.04–3.83)	1.69	(0.23–12.6)	0.88	(0.43–1.81)	N/A		N/A	
CMIT/MIT	0.87	(0.74–1.04)	1.29	(1.09–1.53)	1.08	(0.75–1.56)	1.16	(0.99–1.37)	1.22	(1.01–1.47)	1.22	(0.67–2.25)	0.94	(0.77–1.14)	0.78	(0.51–1.21)	0.83	(0.51–1.34)
Others	0.86	(0.57–1.28)	1.59	(1.01–2.34)	0.95	(0.38–2.37)	1.03	(0.70–1.49)	1.08	(0.70–1.68)	2.87	(1.10–7.38)	0.79	(0.50–1.26)	0.84	(0.30–2.40)	1.17	(0.41–3.30)
Exposure direction (*n* = 4,145)																		
Toward the other sides	1.00	(Reference)	1.00	(Reference)	1.00	(Reference)	1.00	(Reference)	1.00	(Reference)	1.00	(Reference)	1.00	(Reference)	1.00	(Reference)	1.00	(Reference)
Toward the face	1.08	(0.93–1.25)	1.03	(0.89–1.20)	1.04	(0.75–1.44)	1.05	(0.91–1.20)	1.00	(0.84–1.19)	1.51	(0.83–2.73)	1.07	(0.90–1.26)	0.93	(0.66–1.30)	0.98	(0.68–1.43)
Exposure proximity (meter) (*n* = 4,142)																		
≥ 1 m	1.00	(Reference)	1.00	(Reference)	1.00	(Reference)	1.00	(Reference)	1.00	(Reference)	1.00	(Reference)	1.00	(Reference)	1.00	(Reference)	1.00	(Reference)
0.5 to < 1 m	0.90	(0.77–1.07)	0.97	(0.83–1.15)	1.33	(0.91–1.95)	1.18	(1.01–1.38)	1.08	(0.89–1.31)	1.11	(0.61–2.04)	1.13	(0.94–1.37)	1.63	(1.08–2.45)	1.72	(1.09–2.72)
< 0.5 m	1.06	(0.90–1.26)	0.91	(0.76–1.08)	1.40	(0.95–2.06)	1.01	(0.86–1.18)	0.99	(0.81–1.22)	1.09	(0.58–2.05)	0.90	(0.73–1.10)	1.58	(1.04–2.39)	1.66	(1.04–2.64)
*P*-trend	0.460		0.267		0.106		0.934		0.894		0.812		0.243		0.051		0.054	
Exposure duration (month) (*n* = 3,981)																		
< 6 months	1.00	(Reference)	1.00	(Reference)	1.00	(Reference)	1.00	(Reference)	1.00	(Reference)	1.00	(Reference)	1.00	(Reference)	1.00	(Reference)	1.00	(Reference)
6 to < 12 months	1.17	(0.89–1.80)	1.54	(1.14–2.08)	0.94	(0.48–1.85)	1.20	(0.93–1.55)	1.52	(1.08–2.14)	1.12	(0.32–3.85)	1.29	(0.92–1.79)	0.73	(0.33–1.61)	1.00	(0.35–.87)
12 to < 24 months	1.04	(0.80–1.34)	1.81	(1.37–2.40)	1.26	(0.69–2.28)	1.30	(1.03–1.65)	1.54	(1.11–2.12)	1.53	(0.50–4.64)	1.26	(0.93–1.72)	1.48	(0.76–2.87)	2.50	(1.03–6.07)
≥ 24 months	1.24	(0.98–1.58)	2.01	(1.54–2.62)	1.35	(0.77–2.35)	1.46	(1.17–1.82)	1.89	(1.40–2.57)	1.98	(0.70–5.62)	1.57	(1.18–2.11)	1.52	(0.82–2.83)	2.75	(1.18–6.40)
*P*-trend	0.081		< .001		0.110		< .001		< .001		0.074		< .001		0.019		0.001	
Cumulative exposure time (hr)^a^ (*n* = 3,896)																		
Low	1.00	(Reference)	1.00	(Reference)	1.00	(Reference)	1.00	(Reference)	1.00	(Reference)	1.00	(Reference)	1.00	(Reference)	1.00	(Reference)	1.00	(Reference)
Medium	0.97	(0.80–1.18)	1.17	(0.96–1.43)	1.11	(0.93–1.54)	1.11	(0.93–1.33)	1.37	(1.09–1.72)	0.99	(0.43–2.31)	1.22	(0.98–1.53)	1.26	(0.79–2.06)	1.39	(0.79–2.44)
High	1.07	(0.88–1.30)	1.30	(1.07–1.59)	1.31	(0.84–2.03)	1.24	(1.03–1.48)	1.20	(0.95–1.52)	1.54	(0.71–3.30)	1.07	(0.85–1.34)	1.48	(0.93–2.36)	1.85	(1.08–3.16)
Very high	1.25	(1.03–1.52)	1.40	(1.14–1.71)	1.83	(1.21–2.76)	1.51	(1.26–1.82)	1.44	(1.14–1.82)	2.17	(1.05–4.46)	1.54	(1.23–1.93)	1.63	(1.04–2.56)	2.03	(1.21–3.42)
*P*-trend	0.015		0.001		0.001		< .001		0.012		0.014		0.001		0.027		0.004	
Indoor air concentration (µg/m^3^)^b^ (*n* = 2,845)																		
Low	1.00	(Reference)	1.00	(Reference)	1.00	(Reference)	1.00	(Reference)	1.00	(Reference)	1.00	(Reference)	1.00	(Reference)	1.00	(Reference)	1.00	(Reference)
Medium	1.10	(0.88–1.38)	0.81	(0.65–1.02)	0.82	(0.48–1.38)	1.09	(0.88–1.34)	0.97	(0.75–1.25)	0.67	(0.27–1.65)	0.93	(0.72–1.21)	1.07	(0.62–1.85)	1.00	(0.52–1.88)
High	1.10	(0.88–1.38)	0.98	(0.78–1.22)	1.25	(0.78–2.03)	1.16	(0.94–1.44)	0.91	(0.71–1.18)	0.65	(0.27–1.62)	1.19	(0.92–1.53)	1.27	(0.76–2.15)	1.40	(0.78–2.54)
Very high	1.32	(1.05–1.70)	0.92	(0.73–1.15)	1.32	(0.82–2.13)	1.06	(0.86–1.32)	0.80	(0.62–1.04)	0.95	(0.41–2.17)	1.15	(0.90–1.49)	1.13	(0.66–1.94)	1.13	(0.61–2.09)
*P*-trend	0.023		0.837		0.098		0.472		< .001		0.871		0.101		0.526		0.478	
Cumulative exposure level (µg/m^3^ × hr)^c^(*n* = 2,708)																		
Low	1.00	(Reference)	1.00	(Reference)	1.00	(Reference)	1.00	(Reference)	1.00	(Reference)	1.00	(Reference)	1.00	(Reference)	1.00	(Reference)	1.00	(Reference)
Medium	0.91	(0.72–1.15)	0.89	(0.70–1.13)	1.48	(0.86–2.55)	1.50	(1.20–1.86)	1.15	(0.89–1.50)	0.71	(0.22–2.25)	1.07	(0.81–1.41)	1.09	(0.61–1.94)	0.96	(0.48–1.90)
High	1.10	(0.88–1.39)	1.04	(0.82–1.31)	1.42	(0.82–2.46)	1.18	(0.95–1.46)	1.16	(0.89–1.51)	1.27	(0.47–3.44)	1.50	(1.16–1.95)	1.33	(0.76–2.32)	1.31	(0.69–2.49)
Very high	1.30	(1.03–1.63)	1.30	(1.03–1.63)	1.98	(1.18–3.32)	1.59	(1.27–1.98)	1.10	(0.84–1.45)	2.50	(1.02–6.10)	1.55	(1.19–2.01)	1.41	(0.82–2.43)	1.54	(0.83–2.85)
*P*-trend	0.009		0.013		0.014		0.001		0.473		0.014		< .001		0.160		0.095	

Models were adjusted for age, sex, education, BMI, and cigarette smoke

Brain disease: brain and cerebrovascular disease

^a^Classified by quartile cut-points: 2688, 6468, and 14,112 h

^b^Classified by quartile cut-points: 293.04, 493.37, and 866.39 µg/m^3^

^c^Classified by quartile cut-points: 864,130, 2,988,271, and 8,729,147 µg/m^3^ × hr

Table 3 shows results of logistic regression models after further adjustment for other environmental exposures of commercial chemicals, house mold, neighborhood factory or incineration, and occupational exposure that might affect health conditions. When we controlled all four kinds of environmental factors, associations of nine diseases with HD exposures were not changed from results of original models, i.e., Table 2. Results in separate models after controlling for each of environmental factors are presented in Supplementary Materials, Tables S12-S15.

**Table 3:** ORs (95% CIs) of reported health conditions by humidifier disinfectant exposures after further adjustment for environmental exposures

Exposures	Pneumonia		Asthma		Cardiovascular disease		Respiratory disease		Otorhinolaryng-ologic disease		Brain disease		Dermatological disease		All cancers		Lung cancer	
Chemical type (*n* = 3,958)																		
PHMG	1.00	(Reference)	1.00	(Reference)	1.00	(Reference)	1.00	(Reference)	1.00	(Reference)	1.00	(Reference)	1.00	(Reference)	1.00	(Reference)	1.00	(Reference)
PGH	0.81	(0.42–1.55)	0.84	(0.44–1.64)	1.26	(0.30–5.32)	0.74	(0.40–1.35)	1.95	(1.02–3.73)	1.67	(0.22–12.5)	0.88	(0.43–1.84)	N/A		N/A	
CMIT/MIT	0.88	(0.74–1.04)	1.29	(1.09–1.53)	1.06	(0.73–1.54)	1.16	(0.99–1.37)	1.21	(1.00–1.47)	1.25	(0.68–2.30)	0.84	(0.77–1.14)	0.79	(0.51–1.22)	0.88	(0.74–1.04)
Others	0.87	(0.58–1.31)	1.57	(1.07–2.30)	0.95	(0.38–2.39)	1.02	(0.70–1.48)	1.07	(0.69–1.66)	2.91	(1.12–7.56)	0.80	(0.50–1.27)	0.88	(0.32–2.47)	0.87	(0.58–1.31)
Exposure direction (*n* = 4,145)																		
Toward the other sides	1.00	(Reference)	1.00	(Reference)	1.00	(Reference)	1.00	(Reference)	1.00	(Reference)	1.00	(Reference)	1.00	(Reference)	1.00	(Reference)	1.00	(Reference)
Toward the face	1.08	(0.93–1.26)	1.02	(0.88–1.18)	1.07	(0.77–1.49)	1.04	(0.90–1.19)	0.99	(0.84–1.18)	1.47	(0.81–2.66)	1.07	(0.90–1.27)	0.92	(0.66–1.29)	0.98	(0.67–1.43)
Exposure proximity (meter) (*n* = 4,142)																		
≥ 1 m	1.00	(Reference)	1.00	(Reference)	1.00	(Reference)	1.00	(Reference)	1.00	(Reference)	1.00	(Reference)	1.00	(Reference)	1.00	(Reference)	1.00	(Reference)
0.5 to < 1 m	0.90	(0.76– 1.06)	0.97	(0.82–1.15)	1.35	(0.92–1.98)	1.17	(1.01–1.37)	1.07	(0.89–1.30)	1.10	(0.60–2.01)	1.14	(0.94–1.37)	1.62	(1.08–2.43)	1.72	(1.09–2.72)
< 0.5 m	1.06	(0.89–1.27)	0.89	(0.75–1.07)	1.46	(0.99–2.16)	0.99	(0.85–1.17)	0.98	(0.79–1.220	1.05	(0.55–1.98)	0.90	(0.74–1.11)	1.57	(1.04–2.39)	1.66	(1.04–2.65)
*P*-trend	0.416		0.206		0.067		0.819		0.789		0.904		0.278		0.052		0.052	
Exposure duration (month) (*n* = 3,981)																		
< 6 months	1.00	(Reference)	1.00	(Reference)	1.00	(Reference)	1.00	(Reference)	1.00	(Reference)	1.00	(Reference)	1.00	(Reference)	1.00	(Reference)	1.00	(Reference)
6 to < 12 months	1.17	(0.89–1.55)	1.54	(1.14–2.08)	0.93	(0.47–1.83)	1.20	(0.93–1.56)	1.53	(1.08–2.15)	1.16	(0.34–4.00)	1.29	(0.93–1.80)	0.74	(0.33–1.64)	1.01	(0.35–2.90)
12 to < 24 months	1.04	(0.80–1.34)	1.81	(1.37–2.40)	1.23	(0.68–2.24)	1.31	(1.03–1.66)	1.53	(1.11–2.11)	1.52	(0.50–4.61)	1.26	(0.92–1.72)	1.49	(0.77–2.90)	2.51	(1.03–6.10)
≥ 24 months	1.23	(0.97–1.57)	2.03	(1.54–2.65)	1.33	(0.76–2.31)	1.47	(1.18–1.84)	1.91	(1.41–2.60)	2.03	(0.72–5.76)	1.57	(1.17–2.10)	1.53	(0.82–2.83)	2.75	(1.18–6,39)
*P*-trend	0.104		< .001		0.122		< .001		< .001		0.070		0.001		0.021		< .001	
Cumulative exposure time (hr)^a^ (*n* = 3,896)																		
Low	1.00	(Reference)	1.00	(Reference)	1.00	(Reference)	1.00	(Reference)	1.00	(Reference)	1.00	(Reference)	1.00	(Reference)	1.00	(Reference)	1.00	(Reference)
Medium	0.97	(0.80–1.18)	1.16	(0.96–1.41)	0.98	(0.61–1.57)	1.10	(0.92–1.32)	1.37	(1.09–1.72)	0.96	(0.41–2.23)	1.23	(0.98–1.53)	1.27	(0.79–2.06)	1.38	(0.79–2.44)
High	1.07	(0.88–1.29)	1.30	(1.07–1.59)	1.33	(0.85–2.06)	1.23	(1.03–1.48)	1.20	(0.95–1.52)	1.51	(0.70–3.26)	1.07	(0.85–1.35)	1.47	(0.92–2.34)	1.84	(1.07–3.15)
Very high	1.25	(1.03–1.52)	1.40	(1.15–1.71)	1.84	(1.22–2.78)	1.51	(1.26–1.82)	1.44	(1.14–1.82)	2.16	(1.05–4.46)	1.54	(1.23–1.93)	1.62	(1.03–2.54)	2.02	(1.20–3.40)
*P*-trend	0.016		< .001		< .001		< .001		0.012		0.014		0.001		0.029		0.004	
Indoor air concentration (µg/m^3^)^b^ (*n* = 2,845)																		
Low	1.00	(Reference)	1.00	(Reference)	1.00	(Reference)	1.00	(Reference)	1.00	(Reference)	1.00	(Reference)	1.00	(Reference)	1.00	(Reference)	1.00	(Reference)
Medium	1.10	(0.88–1.39)	0.82	(0.65–1.03)	0.81	(0.48–1.38)	1.09	(0.88–1.35)	0.97	(0.75–1.25)	0.68	(0.28–1.69)	0.93	(0.71–1.20)	1.08	(0.62–1.85)	1.00	(0.53–1.90)
High	1.10	(0.88–1.39)	0.97	(0.77–1.21)	1.30	(0.80–2.11)	1.15	(0.93–1.43)	0.90	(0.70–1.17)	0.66	(0.27–1.62)	1.19	(0.92–1.53)	1.27	(0.75–2.14)	1.40	(0.77–2.53)
Very high	1.32	(1.05–1.66)	0.92	(0.73–1.15)	1.32	(0.82–2.13)	1.06	(0.86–1.32)	0.80	(0.62–1.04)	0.95	(0.42–2.18)	1.15	(0.90–1.49)	1.15	(0.67–1.97)	1.14	(0.61–2.12)
*P*-trend	0.021		0.776		0.091		0.492		0.081		0.868		0.095		0.504		0.464	
Cumulative exposure level (µg/m^3^ × hr)^c^(*n* = 2,708)																		
Low	1.00	(Reference)	1.00	(Reference)	1.00	(Reference)	1.00	(Reference)	1.00	(Reference)	1.00	(Reference)	1.00	(Reference)	1.00	(Reference)	1.00	(Reference)
Medium	0.91	(0.72–1.15)	0.89	(0.70–1.13)	1.47	(0.85–2.53)	1.50	(1.21–1.87)	1.16	(0.89–1.51)	0.71	(0.22–2.25)	1.07	(0.82–1.41)	1.11	(0.62–1.98)	0.98	(0.49–1.94)
High	1.10	(0.88–1.39)	1.04	(0.82–1.31)	1.46	(0.84–2.53)	1.17	(0.94–1.45)	1.16	(0.89–1.51)	1.27	(0.47–3.43)	1.50	(1.16–1.95)	1.32	(0.75–2.30)	1.30	(0.68–2.47)
Very high	1.30	(1.03–1.63)	1.30	(1.03–1.64)	2.05	(1.22–3.44)	1.58	(1.27–1.98)	1.10	(0.84–1.44)	2.53	(1.03–6.17)	1.55	(1.19–2.01)	1.40	(0.81–2.41)	1.52	(0.82–2.83)
*P*-trend	0.010		0.013		0.008		0.002		0.481		0.014		< .001		0.183		0.109	

Models were adjusted for covariates in Table 2 and additionally adjusted for environmental exposures of commercial chemical, house mold, neighborhood factory or incineration, and occupational exposure

Brain disease: brain and cerebrovascular disease

^a^Classified by quartile cut-points: 2688, 6468, and 14,112 h

^b^Classified by quartile cut-points: 293.04, 493.37, and 866.39 µg/m^3^

^c^Classified by quartile cut-points: 864,130, 2,988,271, and 8,729,147 µg/m^3^ × hr

## Discussion

Our study evaluated the association between HD exposure and the prevalence of various health conditions in claimants for compensation using personal HD exposure assessment data. After adjustment for potential confounding factors, HD exposure was significantly associated with the increased risk of all nine reported health conditions: pneumonia, asthma, cardiovascular disease, respiratory disease, otorhinolaryngologic disease, brain disease, dermatological disease, lung cancer, and all cancers. These associations did not alter even after further controlling for environmental and occupational exposures to chemicals.

Although health problems caused by HD exposures may include various organs and its etiology is poorly understood, its main underlying mechanisms are reactive oxygen species and inflammation that can lead to cell deaths and genomic alterations [21, 22]. Chemical components in HD commonly attack cell membranes in different ways, PHMG induces the inhibition of dehydrogenase activity, resulting in cell membrane damage, PGH acts as a β-lactamase inhibitor and destroys the physical structure of the cell membrane, and CMIT/MIT inhibits the activity of enzymes with sulfhydryl group (-SH) and other proteins, leading to cell death [23]. Together, chemical components in HD commonly initiate biological pathways known to induce increment of reactive oxygen species (ROS) and generation of inflammatory responses, which can result in the loss of protein function and cell deaths [24, 25]. According to previous epidemiological studies, ROS and inflammation can promote both allergic inflammation and systemic chronic inflammation [26, 27] that contribute to the development of allergic diseases such as atopic dermatitis [28] and allergic rhinitis [29], inflammatory diseases such as pneumonia [23], asthma and respiratory disease [30]. Furthermore, several *in vivo* studies have investigated adverse effects of HD chemical components on various health conditions. Zebrafish with treatment of PHMG and PGH have high levels of production of ROS and severe inflammation with induced damages in heart [12], mice after intratracheal installation of PHMG show airway inflammation and asthmatic features [19], mice with epicutaneous exposure to CMIT/MIT show enhanced atopic dermatitis symptoms [13], and rat with an inhalation exposure to PHMG shows alteration in tumor suppressor gene that may exacerbate carcinogenesis due to its functional loss [25]. Considering the spread of HD to the whole body, the toxicity of PGH, PHMG, and CMIT/MIT may promote ROS and inflammation, leading to adverse effects on various organs besides lung injury.

In the current study, we evaluated exposures of HD using various models: chemical type, exposure direction, exposure proximity, exposure duration, cumulative exposure time, indoor air concentration, and cumulative exposure level. Interestingly, all nine reported health conditions analyzed in the current study were observed to have significant dose-response associations with cumulative exposure time, while most of these health conditions were not associated with indoor air concentration. Our observations indicate that long-term cumulative exposures to HDs even in low levels might be toxic with potential effects various organs. Although acute exposure might be the key to lung injury, which is still of debate, our findings suggest that long-term exposure may be the determining factor in diseases other than lung injury. Interestingly, exposure proximity (<1 m vs. ≥ 1 m) was observed to have significant associations only with lung cancer and all cancers in the current study. However, the scientific evidence with different models of HD exposure characteristics is still lacking, thus, future studies in relation to various target organs are warranted.

Also, the current study evaluated chemical type of HD products, and CMIT/MIT-containing HD (vs. PHMG-containing HD, the most commonly used chemical) showed significant associations with an increased risk of asthma and otorhinolaryngologic disease. In addition, PGH-containing HD (vs. PHMG-containing HD) presented significant associations with the risk of otorhinolaryngologic disease. A few epidemiologic studies have evaluated adverse effects of certain HD chemicals on several diseases. A recent study using a Panel Study of Korean Children (PSKC) data has shown that participants exposed to PHMG/PGH-containing HD compared to those never exposed to HD have a significantly increased risk of otorhinolaryngologic disease [16]. In a case report, patients after exposure to only CMIT/MIT-containing HD had allergic reactions and decreased lung functions that resulted in asthma [31]. However, it is difficult to directly compare our findings with previous studies because our study compared different chemicals’ usages to each other (PGH, CMIT/MIT, others, vs. PHMG), while other studies compared certain chemical groups with non-usage group. Nevertheless, our findings suggest that CMIT/MIT or PGH-containing HDs might increase risks for certain diseases, although PHMG-containing HDs are well-known to be associated with lung injury. Observed associations in the present study remained statistically significant even after further controlling for environmental and occupational exposures, i.e., each of household chemical usage, house mold, neighborhood factory or incineration, workplace exposure to toxic gas, fumes, or smoke, which might induce adverse health effects on various diseases. Our findings were consistent with findings of a previous study of children, which suggested that the association between HD exposure and allergic rhinitis did not alter even after adjusting for environmental exposures of passive smoking, presence of house mold, or presence of pet cats or dogs [16].

When the current study stratified by children-to-adolescents and adults, HD exposure presented significant associations with asthma and dermatological disease in both age groups (stronger in children-to-adolescents than adults), pneumonia and otorhinolaryngologic disease only in children-to-adolescents, and respiratory disease only in adults. Their discussion is available in the Supplementary Material, “Age-stratified Analysis: Discussion”.

The main strengths of our study are as follows. First, we used well-designed personal exposure assessment data, which included information on a variety of HD exposure-related questionnaires and adverse health effects. Second, we considered the adjustment for other environmental factors such as commercial chemicals, house mold, neighboring factories or incinerators, and occupational exposure in a sensitivity analysis because environmental factors might also affect health conditions. Nevertheless, our study also has some limitations. First, there was a potential recall bias because HD exposure assessment was based on the subject’s responses for HD use in the past. In spite of well-structured interviews including logical and repetitive questions, measurement errors due to recall bias could remain. Second, due to the nature of the current population that all subjects had exposure to HD and at least one health conditions as potential victims were enrolled in governmental compensation, we could infer an inevitable but critical limitation of selection bias, which could result in the lack of generalizability as well as potential over-estimates in exposure assessment. Nevertheless, those overestimates might be non-differential in whole population regardless of the presence of each of the nine diseases. Third, we could not rule out the possibility that health outcomes included in this study might have affected each other, which might potentially act as intermediaries, in part, for each other. Fourth, health outcomes defined by self-reported physician’s diagnosis could not exclude a potential misclassification because their health outcomes were not medically confirmed at the time of data collection. Finally, although subjects reported ‘health conditions after HD exposure’, information about exposure and outcome were collected at the same time. Thus, we could not infer causal association in our findings.

## Conclusions

The current study suggests that cumulative exposures to HD might be associated with higher risks of various health outcomes of pneumonia, asthma, cardiovascular disease, respiratory disease, otorhinolaryngologic disease, brain disease, dermatological disease, and cancer. In addition to the well-known evidence in lung injury, our findings show a necessity of epidemiological as well as toxicological research that HD exposures could lead to adverse effects on various organs.

### Supplementary Information

**Additional file 1: **Age-stratified Analysis: Methods. Age-stratified Analysis: Results. Age-stratified Analysis: Discussion. **Table S1.** Classification of reported health conditions.  **Table S2.** Participants’ distribution (No. (%)) by pneumonia. **Table S3.** Participants’ distribution (No. (%)) by asthma. **Table S4.** Participants’ distribution (No. (%)) by cardiovascular disease. **Table S5.** Participants’ distribution (No. (%)) by respiratory disease. **Table S6.** Participants’ distribution (No. (%)) by otorhinolaryngologic disease. **Table S7.** Participants’ distribution (No. (%)) by brain disease. **Table S8.** Participants’ distribution (No. (%)) by dermatological disease. **Table S9.** Participants’ distribution (No. (%)) by all cancers. **Table S10.** Participants’ distribution (No. (%)) by lung cancer. **Table S11.** Participants’ exposure characteristics in overall subjects and subjects with each of nine comorbid health conditions. **Table S12.** ORs (95% CIs) of comorbid health conditions by humidifier disinfectant exposures after further adjustment for commercial chemicals. **Table S13.** ORs (95% CIs) of comorbid health conditions by humidifier disinfectant exposures after further adjustment for house mold. **Table S14.** ORs (95% CIs) of comorbid health conditions by humidifier disinfectant exposures after further adjustment for neighborhood factory or incineration. **Table S15.** ORs (95% Cis) of comorbid health conditions by humidifier disinfectant exposures after further adjustment for occupational exposure. **Figure S1.** ORs (95% CIs) of various diseases by humidifier disinfectant exposures in the age group of < 20 and ≥ 20 years. 

## Data Availability

The datasets analyzed during the current study are available from the corresponding author on reasonable request.

## Abbreviations

HD: Humidifier disinfectants
PHMG: Polyhexamethylene guanidine
PGH: Oligo-(2-(2-ethoxy)-ethoxyethyl) guanidine
CMIT: Chloromethylisothiazolinone
MIT: Methylisothiazolinone
HDLIs: Humidifier disinfectant-associated lung injuries
