# Supplementary material for: Exposures to humidifier disinfectant and various health conditions in Korean based on personal exposure assessment data of claimants for compensation
Source: BMC Public Health. 2023 Oct 2;23:1800. doi: 10.1186/s12889-023-16389-x (PMC10544189; doi:10.1186/s12889-023-16389-x)
Supplement: Supplementary file 1 — Additional file 1: Age-stratified Analysis: Methods. Age-stratified Analysis: Results. Age-stratified Analysis: Discussion. Table S1. Classification of reported health conditions. Table S2. Participants’ distribution (No. (%)) by pneumonia. Table S3. Participants’ distribution (No. (%)) by asthma. Table S4. Participants’ distribution (No. (%)) by cardiovascular disease. Table S5. Participants’ distribution (No. (%)) by respiratory disease. Table S6. Participants’ distribution (No. (%)) by otorhinolaryngologic disease. Table S7. Participants’ distribution (No. (%)) by brain disease. Table S8. Participants’ distribution (No. (%)) by dermatological disease. Table S9. Participants’ distribution (No. (%)) by all cancers. Table S10. Participants’ distribution (No. (%)) by lung cancer. Table S11. Participants’ exposure characteristics in overall subjects and subjects with each of nine comorbid health conditions. Table S12. ORs (95% CIs) of comorbid health conditions by humidifier disinfectant exposures after further adjustment for commercial chemicals. Table S13. ORs (95% CIs) of comorbid health conditions by humidifier disinfectant exposures after further adjustment for house mold. Table S14. ORs (95% CIs) of comorbid health conditions by humidifier disinfectant exposures after further adjustment for neighborhood factory or incineration. Table S15. ORs (95% Cis) of comorbid health conditions by humidifier disinfectant exposures after further adjustment for occupational exposure. Figure S1. ORs (95% CIs) of various diseases by humidifier disinfectant exposures in the age group of < 20 and ≥ 20 years. [file 12889_2023_16389_MOESM1_ESM.pdf]

Supplementary Materials

Exposures to Humidifier Disinfectant and Various Health Conditions in Korean Based on Personal Exposure Assessment Data of Claimants for Compensation

Contents

Supplementary Materials, Text.

- **Age-stratified Analysis: Methods.**
- **Age-stratified Analysis: Results.**
- **Age-stratified Analysis: Discussion.**

Supplementary Materials, Tables.

- **Table S1. Classification of reported health conditions.**
- **Table S2. Participants' distribution (No. (%)) by pneumonia.**
- **Table S3. Participants' distribution (No. (%)) by asthma.**
- **Table S4. Participants' distribution (No. (%)) by cardiovascular disease.**
- **Table S5. Participants' distribution (No. (%)) by respiratory disease.**
- **Table S6. Participants' distribution (No. (%)) by otorhinolaryngologic disease.**
- **Table S7. Participants' distribution (No. (%)) by brain disease.**
- **Table S8. Participants' distribution (No. (%)) by dermatological disease.**
- **Table S9. Participants' distribution (No. (%)) by all cancers.**
- **Table S10. Participants' distribution (No. (%)) by lung cancer.**
- **Table S11. Participants' exposure characteristics in overall subjects and subjects with each of nine comorbid health conditions.**
- **Table S12. ORs (95% CIs) of comorbid health conditions by humidifier disinfectant exposures after further adjustment for commercial chemicals.**
- **Table S13. ORs (95% CIs) of comorbid health conditions by humidifier disinfectant exposures after further adjustment for house mold.**
- **Table S14. ORs (95% CIs) of comorbid health conditions by humidifier disinfectant exposures after further adjustment for neighborhood factory or incineration.**
- **Table S15. ORs (95% Cis) of comorbid health conditions by humidifier disinfectant exposures after further adjustment for occupational exposure.**
- **Figure S1. ORs (95% CIs) of various diseases by humidifier disinfectant exposures in the age group of < 20 and ≥ 20 years.**

Supplementary Materials, REFERENCE.

Supplementary Materials, Text.

Age-stratified Analysis: Methods.

Another sensitivity analysis was conducted with age-stratified models (< 20 and ≥ 20 years of age) because the association between HD exposures and disease development might differ between children-to-adolescents and adults. The number of subjects who had diseases in a subgroup was too small for reliable statistical analysis (cardiovascular disease, n = 31; brain disease, n = 21; all cancers, n = 5; and lung cancer, n = 3) in the children-to-adolescent group.

Age-stratified Analysis: Results.

Figure 3 presents results of associations between comorbid health conditions and HD exposures in subgroups stratified by age (i.e., children-to-adolescents and adults). Pneumonia and otorhinolaryngologic disease showed significant associations with cumulative exposure level and cumulative exposure time only in children-to-adolescents (p for trend: = 0.034 and = 0.018, respectively), but not in adults with any exposure characteristics. Respiratory disease showed significant associations with cumulative exposure time and cumulative exposure level only in adults (both p for trend: < 0.001). Asthma and dermatological diseases showed significant associations with cumulative exposure level and/or cumulative exposure time in both age groups, with stronger associations in children-to-adolescents than in adults, although no disease showed significant interaction by age groups (all p for interaction > 0.05).

Age-stratified Analysis: Discussion.

Because the prevalence and biological susceptibility of certain diseases may differ depending on age, the current study examined models in subgroups of children-to-adolescents and adults. Pneumonia and otorhinolaryngologic disease showed significant dose-dependent associations with an increased exposure to HD only in children-to-adolescents, while respiratory disease showed significant dose-dependent associations with an increased exposure to HD only in adults. In addition, asthma and dermatological disease showed significant dose-dependent associations with an increased exposure to HD in both age groups, but stronger in children-to-adolescents. In general, asthma, allergic rhinitis, and atopic dermatitis are more prevalent in children-to-adolescents than in adults.[1] Although few investigations have evaluated associations between an increased HD exposure and diseases mentioned above, several epidemiological studies have compared HD used children with unused children and reported broadly consistent results, i.e., higher risks of asthma, allergic and dermatological disease in HD used children than in unused children. For example, a Korean children study using PSKC data found a higher risk of allergic rhinitis in children who had used HD in the past than in those who had never used HD.[2] A cross-sectional study using the Korean National Health and Nutrition Examination Survey (KNHANES) suggested the inhalation of HD might be a possible reason for the higher prevalence of asthma and atopic dermatitis in preschool children between 2008 and 2009 compared to other years.[3] Taken together, exposure to HD chemicals in children might make them become more vulnerable to asthma, otorhinolaryngologic

diseases, and dermatological disease than in adults in terms of biological susceptibility. To the best of our knowledge, few epidemiological studies have investigated associations between HD exposures and diseases mentioned above in adults.

Table S1. Classification of reported health condition.

Health condition	Subtypes
Pneumonia	Acute pneumonia, interstitial pneumonia, eosinophilic pneumonia, hypersensitivity pneumonitis, idiopathic interstitial pneumonia, croupous pneumonia, septic pneumonia, aspiration pneumonia, viral pneumonia, bronchopneumonia, and pneumonia of unknown cause
Asthma	Pediatric asthma, rapid progression asthma attack, allergic asthma, bronchial asthma, and atopic asthma
Cardiovascular disease	Hypertension, heart failure, myocardial infarction, thrombocytopenia, angina, peripheral vascular disease, kawasaki disease, dyslipidemia, thrombosis, and atherosclerosis
Respiratory disease	Apnea, respiratory failure, tracheal wall hypertrophy, bronchiectasia, bronchiolitis obliterans, acute bronchitis, respiratory disorders, tuberculosis, pulmonary emphysema, pulmonary fibrosis, pneumothorax, allergic respiratory disease, respiratory syndrome, hyperventilation syndrome, and tracheal stenosis
Otorhinolaryngologic disease	Rhinitis, hearing loss, rhinosinusitis, sinusitis, anosmia, tinnitus, cervical lymphadenitis, tonsillitis, laryngopharyngitis, otitis media, vestibulitis, hypertrophy of adenoids, meniere's disease, nasal septal deviation, nodules of vocal cord and larynx, hyposmia, and postnasal drip
Brain disease	Dementia, cerebral palsy, epilepsy, brain abscess, pyocephalus, brain death, hyperpituitarism, encephalomeningitis, brain damage, brain tumor, encephalitis, hemisphericity, attention-deficit hyperactivity disorder (ADHD), developmental disability, seizure, and intellectual disability, cerebral stroke, cerebral small vessel disease, occlusion of cerebral artery, brain hemorrhage, and brain infarction
Dermatological disease	Atopic dermatitis, dermatitis, psoriasis, urticaria, herpes zoster, pruritus, eczema, and seborrheic dermatitis
All cancers	Lung cancer, thyroid cancer, liver cancer, laryngeal cancer, nasal cavity cancer, lymphoma, breast cancer, renal cancer, colon cancer, tongue cancer, stomach cancer, peritoneal cancer, skin cancer, cervical cancer, rectal cancer, thymic cancer, and leukemia
Lung cancer	Lung cancer, adenocarcinoma of the lung, small cell lung carcinoma, and non-small cell lung cancer

Brain disease: brain and cerebrovascular disease.

Table S2. Participants' distribution (No. (%)) by pneumonia.

Characteristics	Pneumonia (n = 1,484)	Not pneumonia (n = 2,695)	*P*-value[a]
BMI [kg/m²]			<.001
Low (< 23)	1,077 (72.6)	1,614 (59.9)	
High (≥ 23)	407 (27.4)	1,081 (40.1)	
Age [years]			<.001
< 10	720 (48.5)	571 (21.2)	
10~19	27 (1.8)	88 (3.3)	
20~29	70 (4.7)	213 (7.9)	
30~39	188 (12.7)	528 (19.6)	
40~49	109 (7.4)	382 (14.2)	
50~59	153 (10.3)	359 (13.3)	
60~69	120 (8.1)	366 (13.6)	
70~79	83 (5.6)	157 (5.8)	
≥ 80	14 (0.9)	31 (1.1)	
Sex			0.781
Male	767 (51.7)	1,405 (52.1)	
Female	717 (48.3)	1,290 (47.9)	
Survival status			0.903
Survivors	1,243 (83.8)	2,252 (83.6)	
Non-survivors	241 (16.2)	443 (16.4)	
Cigarette smoking			<.001
Never smoker	1,225 (82.5)	1,939 (72.0)	
Former smoker	225 (15.2)	631 (23.4)	
Current smoker	34 (2.3)	125 (4.6)	
Education			<.001
< High school	866 (58.4)	1,003 (37.2)	
High school	282 (19.0)	738 (27.4)	
> High school	336 (22.6)	954 (35.4)	
Environmental exposures			
Commercial chemicals[b]	4.0 ± 2.3	3.9 ± 2.3	0.316
House mold	89 (6.0)	166 (6.2)	0.834
Neighborhood factory or incineration	24 (1.6)	63 (2.3)	0.119
Occupational exposure	13 (0.9)	46 (1.7)	0.029

Data in tables are presented as mean ± SE for continuous variables and sample size (percentage) for categorical variables.

[a] *P*-value is based on the chi-square test for categorical variables and t-test for continuous variables.

[b] Number of chemicals usage: fabric brightener, household insecticides, deodorant or air freshener, water repellent, disinfectant or sanitizer, paint additive, glass cleaner, mothball, glue, stain remover, dish washing detergent, laundry detergent, scented candle or fumigant, hair styling product, at-home dry cleaner, and polish (range 0–16).

Table S3. Participants' distribution (No. (%)) by asthma.

Characteristics	Asthma (n = 1,301)	Not asthma (n = 2,878)	*P*-value[a]
BMI [kg/m²]			0.045
Low (< 23)	867 (66.6)	1,824 (63.4)	
High (≥ 23)	434 (33.4)	1,054 (36.6)	
Age [years]			<.001
< 10	564 (43.3)	727 (25.3)	
10~19	34 (2.6)	81 (2.8)	
20~29	86 (6.6)	197 (6.8)	
30~39	203 (15.6)	513 (17.8)	
40~49	146 (11.2)	345 (12.0)	
50~59	122 (9.4)	390 (13.6)	
60~69	97 (7.5)	389 (13.5)	
70~79	45 (3.5)	195 (6.8)	
≥ 80	4 (0.3)	41 (1.4)	
Sex			0.127
Male	699 (53.7)	1,473 (51.2)	
Female	602 (46.3)	1,405 (48.8)	
Survival status			<.001
Survivors	1,218 (93.6)	2,277 (79.1)	
Non-survivors	83 (6.4)	601 (20.9)	
Cigarette smoking			<.001
Never smoker	1,043 (80.2)	2,121 (73.7)	
Former smoker	213 (16.4)	643 (22.3)	
Current smoker	45 (3.4)	114 (4.0)	
Education			<.001
< High school	659 (50.7)	1,210 (42.0)	
High school	293 (22.5)	727 (25.3)	
> High school	349 (26.8)	941 (32.7)	
Environmental exposures			
Commercial chemicals[b]	3.9 ± 2.2	4.0 ± 2.3	0.036
House mold	84 (6.5)	171 (5.9)	0.520
Neighborhood factory or incineration	31 (2.4)	56 (2.0)	0.360
Occupational exposure	17 (1.3)	42 (1.5)	0.699

Data in tables are presented as mean ± SE for continuous variables and sample size (percentage) for categorical variables.

[a] *P*-value is based on the chi-square test for categorical variables and t-test for continuous variables.

[b] Number of chemicals usage: fabric brightener, household insecticides, deodorant or air freshener, water repellent, disinfectant or sanitizer, paint additive, glass cleaner, mothball, glue, stain remover, dish washing detergent, laundry detergent, scented candle or fumigant, hair styling product, at-home dry cleaner, and polish (range 0–16).

Table S4. Participants' distribution (No. (%)) by cardiovascular disease.

Characteristics	Cardiovascular disease (n = 203)	Not cardiovascular disease (n = 3,976)	*P*-value[a]
BMI [kg/m²]			0.001
Low (< 23)	108 (53.2)	2,583 (65.0)	
High (≥ 23)	95 (46.8)	1,393 (35.0)	
Age [years]			<.001
< 10	27 (13.3)	1,264 (31.8)	
10~19	4 (2.0)	111 (2.8)	
20~29	9 (4.4)	274 (6.9)	
30~39	31 (15.3)	685 (17.2)	
40~49	40 (19.7)	451 (11.3)	
50~59	45 (22.2)	467 (11.8)	
60~69	32 (15.7)	454 (11.4)	
70~79	12 (5.9)	228 (5.7)	
≥ 80	3 (1.5)	42 (1.1)	
Sex			0.720
Male	108 (53.2)	2,064 (51.9)	
Female	95 (46.8)	1,912 (48.1)	
Survival status			0.305
Survivors	164 (80.8)	3,331 (83.8)	
Non-survivors	39 (19.2)	645 (16.2)	
Cigarette smoking			0.001
Never smoker	132 (65.0)	3,032 (76.3)	
Former smoker	60 (29.6)	796 (20.0)	
Current smoker	11 (5.4)	148 (3.7)	
Education			<.001
< High school	70 (34.5)	1,799 (45.2)	
High school	70 (34.5)	950 (23.9)	
> High school	63 (31.0)	1,227 (30.9)	
Environmental exposures			
Commercial chemicals[b]	4.3 ± 2.4	4.0 ± 2.3	0.022
House mold	15 (7.4)	240 (6.0)	0.432
Neighborhood factory or incineration	4 (2.0)	83 (2.1)	0.909
Occupational exposure	6 (3.0)	53 (1.3)	0.056

Data in tables are presented as mean ± SE for continuous variables and sample size (percentage) for categorical variables.

[a] *P*-value is based on chi-square test for categorical variables and t-test for continuous variables.

[b] Number of chemicals usage: fabric brightener, household insecticides, deodorant or air freshener, water repellent, disinfectant or sanitizer, paint additive, glass cleaner, mothball, glue, stain remover, dish washing detergent, laundry detergent, scented candle or fumigant, hair styling product, at-home dry cleaner, and polish (range 0–16).

Table S5. Participants' distribution (No. (%)) by respiratory disease.

Characteristics	Respiratory disease (n = 2,369)	Not respiratory disease (n = 1,810)	P-value[a]
BMI [kg/m²]			<.001
Low (< 23)	1,590 (67.1)	1,101 (60.8)	
High (≥ 23)	779 (32.9)	709 (39.2)	
Age [years]			<.001
< 10	837 (35.3)	454 (25.1)	
10~19	62 (2.6)	53 (2.9)	
20~29	163 (6.9)	120 (6.6)	
30~39	418 (17.7)	298 (16.5)	
40~49	263 (11.1)	228 (12.6)	
50~59	268 (11.3)	244 (13.5)	
60~69	241 (10.2)	245 (13.5)	
70~79	105 (4.4)	135 (7.5)	
≥ 80	12 (0.5)	33 (1.8)	
Sex			0.063
Male	1,261 (53.2)	911 (50.3)	
Female	1,108 (46.8)	899 (49.7)	
Survival status			<.001
Survivors	2,101 (88.7)	1,394 (77.0)	
Non-survivors	268 (11.3)	416 (23.0)	
Cigarette smoking			0.010
Never smoker	1,835 (77.4)	1,329 (73.4)	
Former smoker	449 (19.0)	407 (22.5)	
Current smoker	85 (3.6)	74 (4.1)	
Education			0.025
< High school	1,100 (46.4)	769 (42.5)	
High school	549 (23.2)	471 (26.0)	
> High school	720 (30.4)	570 (31.5)	
Environmental exposures			
Commercial chemicals[b]	3.9 ± 2.3	4.0 ± 2.3	0.107
House mold	135 (5.7)	120 (6.6)	0.213
Neighborhood factory or incineration	51 (2.2)	36 (2.0)	0.713
Occupational exposure	30 (1.3)	29 (1.6)	0.362

Data in tables are presented as mean ± SE for continuous variables and sample size (percentage) for categorical variables.

[a] P-value is based on chi-square test for categorical variables and t-test for continuous variables.

[b] Number of chemicals usage: fabric brightener, household insecticides, deodorant or air freshener, water repellent, disinfectant or sanitizer, paint additive, glass cleaner, mothball, glue, stain remover, dish washing detergent, laundry detergent, scented candle or fumigant, hair styling product, at-home dry cleaner, and polish (range 0–16).

Table S6. Participants' distribution (No. (%)) by otorhinolaryngologic disease.

Characteristics	Otorhinolaryngologic disease (n = 915)	Not otorhinolaryngologic disease (n = 3,264)	*P*-value[a]
BMI [kg/m²]			<.001
Low (< 23)	648 (70.8)	2,043 (62.6)	
High (≥ 23)	267 (29.2)	1,221 (37.4)	
Age [years]			<.001
< 10	472 (51.6)	819 (25.1)	
10~19	38 (4.2)	77 (2.4)	
20~29	73 (8.0)	210 (6.4)	
30~39	172 (18.8)	544 (16.7)	
40~49	94 (10.3)	397 (12.2)	
50~59	37 (4.0)	475 (14.5)	
60~69	23 (2.5)	463 (14.2)	
70~79	4 (0.4)	236 (7.2)	
≥ 80	2 (0.2)	43 (1.3)	
Sex			0.057
Male	501 (54.8)	1,671 (51.2)	
Female	414 (45.2)	1,593 (48.8)	
Survival status			<.001
Survivors	902 (98.6)	2,593 (79.4)	
Non-survivors	13 (1.4)	671 (20.6)	
Cigarette smoking			<.001
Never smoker	781 (85.4)	2,383 (73.0)	
Former smoker	108 (11.8)	748 (22.9)	
Current smoker	26 (2.8)	133 (4.1)	
Education			<.001
< High school	475 (51.9)	1,394 (42.7)	
High school	176 (19.2)	844 (25.9)	
> High school	264 (28.9)	1,026 (31.4)	
Environmental exposures			
Commercial chemicals[b]	3.9 ± 2.3	4.0 ± 2.3	0.298
House mold	49 (5.4)	206 (6.3)	0.286
Neighborhood factory or incineration	26 (2.8)	61 (1.9)	0.069
Occupational exposure	9 (1.0)	50 (1.5)	0.214

Data in tables are presented as mean ± SE for continuous variables and sample size (percentage) for categorical variables.

[a] *P*-value is based on chi-square test for categorical variables and t-test for continuous variables.

[b] Number of chemicals usage: fabric brightener, household insecticides, deodorant or air freshener, water repellent, disinfectant or sanitizer, paint additive, glass cleaner, mothball, glue, stain remover, dish washing detergent, laundry detergent, scented candle or fumigant, hair styling product, at-home dry cleaner, and polish (range 0–16).

Table S7. Participants' distribution (No. (%)) by brain disease.

Characteristics	Brain disease (n = 69)	Not brain disease (n = 4,110)	*P*-value[a]
BMI [kg/m²]			0.437
Low (< 23)	48 (69.6)	2,643 (64.3)	
High (≥ 23)	21 (30.4)	1,467 (35.7)	
Age [years]			0.638
< 10	20 (29.0)	1,271 (30.9)	
10~19	1 (1.5)	114 (2.8)	
20~29	3 (4.3)	280 (6.8)	
30~39	16 (23.2)	700 (17.0)	
40~49	6 (8.7)	485 (11.8)	
50~59	10 (14.5)	502 (12.2)	
60~69	9 (13.0)	477 (11.6)	
70~79	2 (2.9)	238 (5.8)	
≥ 80	2 (2.9)	43 (1.1)	
Sex			0.031
Male	27 (39.1)	2,145 (52.2)	
Female	42 (60.9)	1,965 (47.8)	
Survival status			0.795
Survivors	59 (85.5)	3,436 (83.6)	
Non-survivors	10 (14.5)	674 (16.4)	
Cigarette smoking			0.144
Never smoker	57 (82.6)	3,107 (75.6)	
Former smoker	8 (11.6)	848 (20.6)	
Current smoker	4 (5.8)	155 (3.8)	
Education			0.672
< High school	29 (42.0)	1,840 (44.8)	
High school	20 (29.0)	1,000 (24.3)	
> High school	20 (29.0)	1,270 (30.9)	
Environmental exposures			
Commercial chemicals[b]	3.6 ± 2.0	4.0 ± 2.3	0.140
House mold	2 (2.9)	253 (6.2)	0.262
Neighborhood factory or incineration	0 (0)	87 (2.1)	0.222
Occupational exposure	3 (4.4)	56 (1.4)	0.037

Data in tables are presented as mean ± SE for continuous variables and sample size (percentage) for categorical variables.

Brain disease: brain and cerebrovascular disease.

[a] *P*-value is based on chi-square test for categorical variables and t-test for continuous variables.

[b] Number of chemicals usage: fabric brightener, household insecticides, deodorant or air freshener, water repellent, disinfectant or sanitizer, paint additive, glass cleaner, mothball, glue, stain remover, dish washing detergent, laundry detergent, scented candle or fumigant, hair styling product, at-home dry cleaner, and polish (range 0–16).

Table S8. Participants' distribution (No. (%)) by dermatological disease.

Characteristics	Dermatological disease (n = 898)	Not dermatological disease (n = 3,281)	*P*-value[a]
BMI [kg/m²]			0.003
Low (< 23)	616 (68.6)	2,075 (63.2)	
High (≥ 23)	282 (31.4)	1,206 (36.8)	
Age [years]			<.001
< 10	429 (47.8)	862 (26.3)	
10~19	29 (3.2)	86 (2.6)	
20~29	64 (7.1)	21`9 (6.7)	
30~39	134 (14.9)	582 (17.7)	
40~49	105 (11.7)	386 (11.8)	
50~59	67 (7.5)	445 (13.6)	
60~69	52 (5.8)	434 (13.2)	
70~79	17 (1.9)	223 (6.8)	
≥ 80	1 (0.1)	44 (1.3)	
Sex			0.637
Male	473 (52.7)	1,699 (51.8)	
Female	425 (47.3)	1,582 (48.2)	
Survival status			<.001
Survivors	853 (95.0)	2,642 (80.5)	
Non-survivors	45 (5.0)	639 (19.5)	
Cigarette smoking			<.001
Never smoker	734 (81.7)	2,430 (74.1)	
Former smoker	143 (15.9)	713 (21.7)	
Current smoker	21 (2.4)	138 (4.2)	
Education			<.001
< High school	458 (51.0)	1,411 (43.0)	
High school	179 (19.9)	841 (25.6)	
> High school	261 (29.1)	1,029 (31.4)	
Environmental exposures			
Commercial chemicals[b]	4.0 ± 2.3	4.0 ± 2.3	0.140
House mold	54 (6.0)	201 (6.1)	0.262
Neighborhood factory or incineration	17 (1.9)	70 (2.1)	0.222
Occupational exposure	11 (1.2)	48 (1.5)	0.037

Data in tables are presented as mean ± SE for continuous variables and sample size (percentage) for categorical variables.

[a] *P*-value is based on chi-square test for categorical variables and t-test for continuous variables.

[b] Number of chemicals usage: fabric brightener, household insecticides, deodorant or air freshener, water repellent, disinfectant or sanitizer, paint additive, glass cleaner, mothball, glue, stain remover, dish washing detergent, laundry detergent, scented candle or fumigant, hair styling product, at-home dry cleaner, and polish (range 0–16).

Table S9. Participants' distribution (No. (%)) by all cancers.

Characteristics	All cancers (n = 189)	Not all cancers (n = 3,990)	*P*-value[a]
BMI [kg/m²]			0.058
Low (< 23)	110 (58.2)	2,581 (64.7)	
High (≥ 23)	79 (41.8)	1,409 (35.3)	
Age [years]			0.058
< 10	2 (1.1)	1,289 (32.3)	
10~19	3 (1.6)	112 (2.8)	
20~29	4 (2.1)	279 (7.0)	
30~39	32 (16.9)	684 (17.1)	
40~49	33 (17.5)	458 (11.5)	
50~59	39 (20.6)	473 (11.9)	
60~69	46 (24.3)	440 (11.0)	
70~79	26 (13.8)	214 (5.4)	
≥ 80	4 (2.1)	41 (1.0)	
Sex			0.740
Male	96 (50.8)	2,076 (52.0)	
Female	93 (49.2)	1,914 (48.0)	
Survival status			
Survivors	108 (57.1)	3,387 (84.9)	
Non-survivors	81 (42.9)	603 (15.1)	
Cigarette smoking			<.001
Never smoker	112 (59.3)	3,052 (76.5)	
Former smoker	70 (37.0)	786 (19.7)	
Current smoker	7 (3.7)	152 (3.8)	
Education			<.001
< High school	49 (25.9)	1,820 (45.6)	
High school	69 (36.5)	951 (23.8)	
> High school	71 (37.6)	1,219 (30.6)	
Environmental exposures			
Commercial chemicals[b]	4.0 ± 2.4	4.0 ± 2.3	0.947
House mold	9 (4.8)	246 (6.2)	0.431
Neighborhood factory or incineration	1 (0.5)	86 (2.2)	0.126
Occupational exposure	4 (2.1)	55 (1.4)	0.401

Data in tables are presented as mean ± SE for continuous variables and sample size (percentage) for categorical variables.

[a] *P*-value is based on chi-square test for categorical variables and t-test for continuous variables.

[b] Number of chemicals usage: fabric brightener, household insecticides, deodorant or air freshener, water repellent, disinfectant or sanitizer, paint additive, glass cleaner, mothball, glue, stain remover, dish washing detergent, laundry detergent, scented candle or fumigant, hair styling product, at-home dry cleaner, and polish (range 0–16).

Table S10. Participants' distribution (No. (%)) by lung cancer.

Characteristics	Lung cancer (n = 153)	Not lung cancer (n = 4,026)	*P*-value[a]
BMI [kg/m²]			0.058
Low (< 23)	87 (56.9)	2,604 (64.7)	
High (≥ 23)	66 (43.1)	1,422 (35.3)	
Age [years]			<.001
< 10	1 (0.7)	1,290 (32.1)	
10~19	2 (1.3)	113 (2.8)	
20~29	4 (2.6)	279 (6.9)	
30~39	19 (12.4)	697 (17.3)	
40~49	24 (15.7)	467 (11.6)	
50~59	33 (21.6)	479 (11.9)	
60~69	43 (28.1)	443 (11.0)	
70~79	23 (15.0)	217 (5.4)	
≥ 80	4 (2.6)	41 (1.0)	
Sex			0.932
Male	79 (51.6)	2,093 (52.0)	
Female	74 (48.4)	1,933 (48.0)	
Survival status			<.001
Survivors	83 (54.2)	3,412 (84.7)	
Non-survivors	70 (45.8)	614 (15.3)	
Cigarette smoking			<.001
Never smoker	88 (57.5)	3,076 (76.4)	
Former smoker	61 (39.9)	795 (19.7)	
Current smoker	4 (2.6)	155 (3.9)	
Education			<.001
< High school	44 (28.8)	1,825 (45.3)	
High school	54 (35.3)	966 (24.0)	
> High school	55 (35.9)	1,235 (30.7)	
Environmental exposures			
Commercial chemicals[b]	4.0 ± 2.5	4.0 ± 2.3	0.884
House mold	7 (4.6)	248 (6.2)	0.422
Neighborhood factory or incineration	1 (0.7)	86 (2.1)	0.207
Occupational exposure	4 (2.6)	55 (1.4)	0.199

Data in tables are presented as mean ± SE for continuous variables and sample size (percentage) for categorical variables.

[a] *P*-value is based on chi-square test for categorical variables and t-test for continuous variables.
[b] Number of chemicals usage: fabric brightener, household insecticides, deodorant or air freshener, water repellent, disinfectant or sanitizer, paint additive, glass cleaner, mothball, glue, stain remover, dish washing detergent, laundry detergent, scented candle or fumigant, hair styling product, at-home dry cleaner, and polish (range 0–16).

Table S11. Participants' exposure characteristics in overall subjects and subjects with each of nine reported health conditions.

Exposures	Pneumonia	Asthma	Cardiovascular disease	Respiratory disease	Otorhinolaryng-ologic disease	Brain disease	Dermatological disease	All cancers	Lung cancer
Chemical type (n=3,958)	1,413	1,253	193	2,268	899	65	873	176	140
PHMG (n=3,021)	1,088 (77.0)	904 (72.2)	148 (76.7)	1,704 (75.1)	645 (71.7)	45 (69.2)	666 (76.3)	146 (83.0)	115 (82.1)
PGH (n=44)	16 (1.1)	13 (1.0)	2 (1.0)	23 (1.0)	17 (1.9)	1 (1.5)	10 (1.1)	0 (0)	0 (0)
CMIT/MIT (n=775)	270 (19.1)	286 (22.8)	38 (19.7)	472 (20.8)	206 (22.9)	14 (21.6)	173 (19.8)	26 (14.8)	21 (15.0)
Others (n=118)	39 (2.8)	50 (4.0)	5 (2.6)	69 (3.1)	31 (3.5)	5 (7.7)	24 (2.8)	4 (2.2)	4 (2.9)
Exposure direction (n=4,145)	1,472	1,295	201	2,360	907	69	893	189	153
Toward the other side (n=1,156)	408 (27.7)	365 (28.2)	52 (25.9)	658 (27.9)	266 (29.3)	14 (20.3)	250 (28.0)	52 (27.5)	40 (26.1)
Toward the face (n=2,989)	1,064 (72.3)	930 (71.8)	149 (74.1)	1,702 (72.1)	641 (70.7)	55 (79.7)	643 (72.0)	137 (72.5)	113 (73.9)
Exposure proximity (meter) (n=4,142)	1,476	1,294	200	2,358	905	69	891	189	153
≥ 1m (n=1,100)	402 (27.3)	354 (27.4)	42 (21.0)	610 (25.9)	236 (26.1)	17 (24.7)	234 (26.3)	36 (19.0)	28 (18.3)
0.5 to < 1m (n=1,693)	591 (40.0)	546 (42.2)	83 (41.5)	1,010 (42.8)	398 (44.0)	29 (42.0)	405 (45.4)	81 (42.9)	66 (43.1)
< 0.5m (n=134)	483 (32.7)	394 (30.4)	75 (37.5)	738 (31.3)	271 (29.9)	23 (33.3)	252 (28.3)	72 (38.1)	59 (38.6)
Exposure duration (month) (n=3,981)	1,420	1,254	196	2,287	882	64	866	178	143
< 6 months (n=389)	137 (9.6)	84 (6.7)	15 (7.7)	199 (8.7)	62 (7.0)	4 (6.3)	68 (7.8)	12 (6.7)	6 (4.2)
6 to < 12 months (n=627)	238 (16.8)	187 (14.9)	22 (11.2)	352 (15.4)	142 (16.1)	7 (10.9)	137 (15.8)	14 (7.9)	9 (6.3)
12 to < 24 months (n=1,018)	354 (24.9)	338 (27.0)	48 (24.5)	589 (25.8)	231 (26.2)	15 (23.4)	218 (25.2)	44 (24.7)	35 (24.5)
≥ 24 months (n=1,947)	691 (48.7)	645 (51.4)	111 (56.6)	1,147 (50.1)	447 (50.7)	38 (59.4)	443 (51.2)	108 (60.7)	93 (65.0)
Cumulative exposure time (hr)[a] (n=3,896)	1,389	1,229	191	2,242	864	63	850	177	142
Low (n=961)	331 (23.8)	265 (21.6)	37 (19.4)	511 (22.8)	180 (20.8)	11 (17.4)	183 (21.5)	32 (18.1)	22 (15.5)
Medium (n=989)	342 (24.6)	311 (25.3)	36 (18.8)	557 (24.8)	244 (28.3)	11 (17.4)	226 (26.6)	40 (22.6)	30 (21.1)
High (n=978)	354 (25.5)	328 (26.7)	48 (25.1)	573 (25.6)	217 (25.1)	17 (27.1)	200 (23.5)	47 (26.5)	40 (28.2)
Very high (n=968)	362 (26.1)	325 (26.4)	70 (36.7)	601 (26.8)	223 (25.8)	24 (38.1)	241 (28.4)	58 (32.8)	50 (35.2)
Indoor air concentration (μg/m³)[b] (n=2,845)	1,000	922	144	1,674	655	39	644	125	95
Low (n=710)	234 (23.4)	245 (26.6)	32 (22.2)	409 (24.4)	178 (27.2)	12 (30.8)	157 (24.4)	27 (21.6)	20 (21.0)
Medium (n=712)	249 (24.9)	212 (23.0)	27 (18.8)	421 (25.2)	171 (26.1)	8 (20.5)	146 (22.7)	30 (24.0)	231 (22.1)
High (n=712)	244 (24.4)	237 (25.7)	41 (28.4)	430 (25.7)	160 (24.4)	8 (20.5)	172 (26.7)	36 (28.8)	30 (31.6)
Very high (n=711)	273 (27.3)	228 (24.7)	44 (30.6)	414 (24.7)	146 (22.3)	11 (28.2)	169 (26.2)	32 (25.6)	24 (25.3)
Cumulative exposure level (μg/m³×hr)[c] (n=2,708)	960	885	139	1,596	622	38	616	120	91
Low (n=677)	236 (24.6)	219 (24.7)	23 (16.6)	362 (22.7)	152 (24.5)	7 (18.4)	134 (21.7)	23 (19.2)	17 (18.7)
Medium (n=677)	214 (22.3)	186 (22.2)	35 (25.2)	421 (26.4)	157 (25.2)	5 (13.2)	133 (21.6)	27 (22.5)	18 (19.8)
High (n=677)	248 (25.8)	221 (25.0)	33 (23.7)	385 (24.1)	163 (26.2)	9 (23.7)	176 (28.6)	33 (27.5)	25 (27.5)
Very high (n=677)	262 (27.3)	249 (28.1)	48 (34.5)	428 (26.8)	150 (24.1)	17 (44.7)	173 (28.1)	37 (30.8)	31 (34.0)

Brain disease: brain and cerebrovascular disease.

[a] Classified by quartile cut-points: 2688, 6468, and 14112 hr.

[b] Classified by quartile cut-points: 293.04, 493.37, and 866.39 μg/m³.

[c] Classified by quartile cut-points: 864130, 2988271, and 8729147 μg/m³×hr.

Table S12. ORs (95% CIs) of reported health conditions by humidifier disinfectant exposures after further adjustment for commercial chemicals.

Exposures	Pneumonia	Asthma	Cardiovascular disease	Respiratory disease	Otorhinolaryng-ologic disease	Brain disease	Dermatological disease	All cancers	Lung cancer
Chemical type (n=3,958)									
PHMG	1 (Reference)	1 (Reference)	1 (Reference)	1 (Reference)	1 (Reference)	1 (Reference)	1 (Reference)	1 (Reference)	1 (Reference)
PGH	0.85 (0.44, 1.64)	0.74 (0.41, 1.36)	1.23 (0.28, 4.92)	1.22 (0.29, 5.18)	1.97 (1.03, 3.77)	1.62 (0.22, 12.1)	0.89 (0.43, 1.83)	N/A	N/A
CMIT/MIT	1.29 (1.09, 1.53)	1.16 (0.99, 1.37)	1.08 (0.75, 1.56)	1.07 (0.74, 1.55)	1.22 (1.00, 1.47)	1.23 (0.67, 2.26)	0.94 (0.77, 1.14)	0.78 (0.51, 1.21)	1.29 (1.09, 1.53)
Others	1.58 (1.08, 2.31)	1.02 (0.70, 1.48)	0.95 (0.38, 2.37)	0.96 (0.38, 2.40)	1.08 (0.69, 1.67)	2.80 (1.08, 7.27)	0.80 (0.50, 1.27)	0.84 (0.30, 2.36)	1.58 (1.08, 2.31)
Exposure direction (n=4,145)									
Toward the other sides	1 (Reference)	1 (Reference)	1 (Reference)	1 (Reference)	1 (Reference)	1 (Reference)	1 (Reference)	1 (Reference)	1 (Reference)
Toward the face	1.08 (0.94, 1.26)	1.02 (0.88, 1.18)	1.04 (0.75, 1.44)	1.07 (0.77, 1.48)	0.99 (0.84, 1.18)	1.48 (0.82, 2.67)	1.07 (0.91, 1.27)	0.92 (0.66, 1.29)	0.98 (0.67, 1.43)
Exposure proximity (meter) (n=4,142)									
≥ 1m	1 (Reference)	1 (Reference)	1 (Reference)	1 (Reference)	1 (Reference)	1 (Reference)	1 (Reference)	1 (Reference)	1 (Reference)
0.5 to < 1m	0.91 (0.77, 1.07)	0.97 (0.82, 1.15)	1.34 (0.92, 1.97)	1.18 (1.01, 1.38)	1.08 (0.89, 1.31)	1.11 (0.60, 2.02)	1.14 (0.94, 1.37)	1.63 (1.08, 2.44)	1.72 (1.09, 2.72)
< 0.5m	1.07 (0.90, 1.27)	0.89 (0.75, 1.07)	1.44 (0.98, 2.13)	1.01 (0.86, 1.18)	0.98 (0.80, 1.20)	1.06 (0.56, 2.00)	0.90 (0.73, 1.11)	1.57 (1.04, 2.39)	1.66 (1.04, 2.64)
P-trend	0.400	0.201	0.074	0.934	0.802	0.884	0.270	0.052	0.054
Exposure duration (month) (n=3,981)									
< 6 months	1 (Reference)	1 (Reference)	1 (Reference)	1 (Reference)	1 (Reference)	1 (Reference)	1 (Reference)	1 (Reference)	1 (Reference)
6 to < 12 months	1.17 (0.89, 1.55)	1.54 (1.14, 2.09)	0.93 (0.48, 1.83)	1.20 (0.93, 1.55)	1.52 (1.08, 2.15)	1.13 (0.33, 3.90)	1.29 (0.92, 1.79)	0.73 (0.33, 1.62)	1.00 (0.35, 2.87)
12 to < 24 months	1.04 (0.80, 1.34)	1.82 (1.37, 2.40)	1.24 (0.68, 2.26)	1.31 (1.03, 1.66)	1.54 (1.12, 2.12)	1.53 (0.50, 4.66)	1.26 (0.93, 1.72)	1.49 (0.77, 2.88)	2.50 (0.93, 6.08)
≥ 24 months	1.24 (0.98,1.58)	2.03 (1.55, 2.64)	1.33 (0.76, 2.31)	1.47 (1.18, 1.84)	1.91 (1.40, 2.59)	2.02 (0.71, 5.72)	1.57 (1.17, 2.10)	1.53 (0.82, 2.84)	2.75 (1.17, 6.41)
P-trend	0.087	<.001	0.126	<.001	<.001	0.068	0.001	0.019	<.001
Cumulative exposure time (hr)[a] (n=3,896)									
Low	1 (Reference)	1 (Reference)	1 (Reference)	1 (Reference)	1 (Reference)	1 (Reference)	1 (Reference)	1 (Reference)	1 (Reference)
Medium	0.97 (0.80, 1.18)	1.17 (0.96, 1.43)	1.11 (0.93, 1.54)	1.11 (0.93, 1.33)	1.37 (1.09, 1.72)	0.99 (0.43, 2.31)	1.22 (0.98, 1.53)	1.26 (0.79, 2.06)	1.39 (0.79, 2.44)
High	1.07 (0.88, 1.30)	1.30 (1.07, 1.59)	1.31 (0.84, 2.03)	1.24 (1.03, 1.48)	1.20 (0.95, 1.52)	1.54 (0.71, 3.30)	1.07 (0.85, 1.34)	1.48 (0.93, 2.36)	1.85 (1.08, 3.16)
Very high	1.25 (1.03, 1.52)	1.40 (1.14, 1.71)	1.83 (1.21, 2.76)	1.51 (1.26, 1.82)	1.44 (1.14, 1.82)	2.17 (1.05, 4.46)	1.54 (1.23, 1.93)	1.63 (1.04, 2.56)	2.03 (1.21, 3.42)
P-trend	0.015	0.001	0.001	<.001	0.012	0.014	0.001	0.027	0.004
Indoor air concentration ($\mu g/m^3$)[b] (n=2,845)									
Low	1 (Reference)	1 (Reference)	1 (Reference)	1 (Reference)	1 (Reference)	1 (Reference)	1 (Reference)	1 (Reference)	1 (Reference)
Medium	1.10 (0.88, 1.38)	0.81 (0.65, 1.02)	0.82 (0.48, 1.38)	1.09 (0.88, 1.34)	0.97 (0.75, 1.25)	0.67 (0.27, 1.65)	0.93 (0.72, 1.21)	1.07 (0.62, 1.85)	1.00 (0.52, 1.88)
High	1.10 (0.88, 1.38)	0.98 (0.78, 1.22)	1.25 (0.78, 2.03)	1.16 (0.94, 1.44)	0.91 (0.71, 1.18)	0.65 (0.27, 1.62)	1.19 (0.92, 1.53)	1.27 (0.76, 2.15)	1.40 (0.78, 2.54)
Very high	1.32 (1.05, 1.70)	0.92 (0.73, 1.15)	1.32 (0.82, 2.13)	1.06 (0.86, 1.32)	0.80 (0.62, 1.04)	0.95 (0.41, 2.17)	1.15 (0.90, 1.49)	1.13 (0.66, 1.94)	1.13 (0.61, 2.09)
P-trend	0.023	0.837	0.098	0.472	<.001	0.871	0.101	0.526	0.478
Cumulative exposure level ($\mu g/m^3 \times hr$)[c] (n=2,708)									
Low	1 (Reference)	1 (Reference)	1 (Reference)	1 (Reference)	1 (Reference)	1 (Reference)	1 (Reference)	1 (Reference)	1 (Reference)
Medium	0.91 (0.72, 1.15)	0.89 (0.70, 1.13)	1.48 (0.86, 2.55)	1.50 (1.20, 1.86)	1.15 (0.89, 1.50)	0.71 (0.22, 2.25)	1.07 (0.81, 1.41)	1.09 (0.61, 1.94)	0.96 (0.48, 1.90)
High	1.10 (0.88, 1.39)	1.04 (0.82, 1.31)	1.42 (0.82, 2.46)	1.18 (0.95, 1.46)	1.16 (0.89, 1.51)	1.27 (0.47, 3.44)	1.50 (1.16, 1.95)	1.33 (0.76, 2.32)	1.31 (0.69, 2.49)
Very high	1.30 (1.03, 1.63)	1.30 (1.03, 1.63)	1.98 (1.18, 3.32)	1.59 (1.27, 1.98)	1.10 (0.84, 1.45)	2.50 (1.02, 6.10)	1.55 (1.19, 2.01)	1.41 (0.82, 2.43)	1.54 (0.83, 2.85)
P-trend	0.009	0.013	0.014	0.001	0.473	0.014	<.001	0.160	0.095

Note: Models were adjusted for covariates in Table 2 and additionally adjusted for commercial chemicals.
Brain disease: brain and cerebrovascular disease.
[a] Classified by quartile cut-points: 2688, 6468, and 14112 hr.
[b] Classified by quartile cut-points: 293.04, 493.37, and 866.39 $\mu g/m^3$.
[c] Classified by quartile cut-points: 864130, 2988271, and 8729147 $\mu g/m^3 \times hr$.

Table S13. ORs (95% CIs) of reported health conditions by humidifier disinfectant exposures after further adjustment for house mold.

Exposures	Pneumonia	Asthma	Cardiovascular disease	Respiratory disease	Otorhinolaryng-ologic disease	Brain disease	Dermatological disease	All cancers	Lung cancer
Chemical type (n=3,958)									
PHMG	1 (Reference)	1 (Reference)	1 (Reference)	1 (Reference)	1 (Reference)	1 (Reference)	1 (Reference)	1 (Reference)	1 (Reference)
PGH	0.79 (0.41, 1.51)	0.87 (0.45, 1.69)	1.18 (0.28, 4.99)	0.76 (0.41, 1.38)	1.98 (1.04, 3.80)	1.66 (0.22, 12.4)	0.88 (0.42, 1.81)	N/A	N/A
CMIT/MIT	0.87 (0.74, 1.04)	1.29 (1.09, 1.53)	1.07 (0.74, 1.55)	1.16 (0.99, 1.37)	1.22 (1.01, 1.48)	1.24 (0.67, 2.27)	0.94 (0.77, 1.14)	0.79 (0.51, 1.22)	0.87 (0.74, 1.04)
Others	0.86 (0.57, 1.28)	1.59 (1.09, 2.34)	0.94 (0.38, 2.36)	1.03 (0.70, 1.50)	1.08 (0.70, 1.68)	2.88 (1.11, 7.46)	0.79 (0.50, 1.26)	0.85 (0.30, 2.38)	0.86 (0.57, 1.28)
Exposure direction (n=4,145)									
Toward the other sides	1 (Reference)	1 (Reference)	1 (Reference)	1 (Reference)	1 (Reference)	1 (Reference)	1 (Reference)	1 (Reference)	1 (Reference)
Toward the face	1.08 (0.93, 1.25)	1.03 (0.89, 1.20)	1.04 (0.75, 1.44)	1.04 (0.91, 1.20)	1.00 (0.84, 1.18)	1.51 (0.83, 2.73)	1.07 (0.90, 1.26)	0.93 (0.66, 1.30)	0.98 (0.68, 1.43)
Exposure proximity (meter) (n=4,142)									
≥ 1m	1 (Reference)	1 (Reference)	1 (Reference)	1 (Reference)	1 (Reference)	1 (Reference)	1 (Reference)	1 (Reference)	1 (Reference)
0.5 to < 1m	0.90 (0.77, 1.06)	0.97 (0.83, 1.15)	1.34 (0.91, 1.96)	1.18 (1.01, 1.37)	1.08 (0.89, 1.31)	1.10 (0.60, 2.02)	1.13 (0.94, 1.37)	1.62 (1.08, 2.44)	1.72 (1.09, 2.72)
< 0.5m	1.06 (0.89, 1.26)	0.91 (0.76, 1.08)	1.40 (0.95, 2.07)	1.00 (0.85, 1.18)	0.99 (0.80, 1.22)	1.08 (0.57, 2.04)	0.89 (0.73, 1.10)	1.57 (1.04, 2.39)	1.66 (1.04, 2.64)
P-trend	0.462	0.270	0.102	0.918	0.830	0.875	0.241	0.052	0.054
Exposure duration (month) (n=3,981)									
< 6 months	1 (Reference)	1 (Reference)	1 (Reference)	1 (Reference)	1 (Reference)	1 (Reference)	1 (Reference)	1 (Reference)	1 (Reference)
6 to < 12 months	1.17 (0.89, 1,55)	1.54 (1.14, 2.08)	0.93 (0.47, 1.83)	1.20 (0.93, 1.56)	1.52 (1.08, 2.15)	1.13 (0.33, 3.89)	1.29 (0.93, 1.79)	0.74 (0.33, 1.63)	1.01 (0.35, 2.90)
12 to < 24 months	1.04 (0.80, 1.34)	1.81 (1.37, 2.39)	1.25 (0.69, 2.26)	1.31 (1.03, 1.66)	1.53 (1.11, 2.11)	1.53 (0.50, 4.64)	1.26 (0.93, 1.72)	1.49 (0.77, 2.89)	2.51 (1.04, 6.11)
≥ 24 months	1.24 (0.98,1.58)	2.01 (1.54, 2.62)	1.34 (0.77, 2.35)	1.46 (1.17, 1.82)	1.89 (1.39, 2.57)	1.98 (0.70, 5.61)	1.57 (1.18, 2.11)	1.53 (0.82, 2.84)	2.75 (1.18, 6.41)
P-trend	0.081	<.001	0.107	<.001	<.001	0.076	0.001	0.020	<.001
Cumulative exposure time (hr)[a] (n=3,896)									
Low	1 (Reference)	1 (Reference)	1 (Reference)	1 (Reference)	1 (Reference)	1 (Reference)	1 (Reference)	1 (Reference)	1 (Reference)
Medium	0.97 (0.80, 1.18)	1.17 (0.96, 1.43)	1.11 (0.93, 1.54)	1.11 (0.93, 1.33)	1.37 (1.09, 1.72)	0.99 (0.43, 2.31)	1.22 (0.98, 1.53)	1.26 (0.79, 2.06)	1.39 (0.79, 2.44)
High	1.07 (0.88, 1.30)	1.30 (1.07, 1.59)	1.31 (0.84, 2.03)	1.24 (1.03, 1.48)	1.20 (0.95, 1.52)	1.54 (0.71, 3.30)	1.07 (0.85, 1.34)	1.48 (0.93, 2.36)	1.85 (1.08, 3.16)
Very high	1.25 (1.03, 1.52)	1.40 (1.14, 1.71)	1.83 (1.21, 2.76)	1.51 (1.26, 1.82)	1.44 (1.14, 1.82)	2.17 (1.05, 4.46)	1.54 (1.23, 1.93)	1.63 (1.04, 2.56)	2.03 (1.21, 3.42)
P-trend	0.015	0.001	0.001	<.001	0.012	0.014	0.001	0.027	0.004
Indoor air concentration (µg/m³)[b] (n=2,845)									
Low	1 (Reference)	1 (Reference)	1 (Reference)	1 (Reference)	1 (Reference)	1 (Reference)	1 (Reference)	1 (Reference)	1 (Reference)
Medium	1.10 (0.88, 1.38)	0.81 (0.65, 1.02)	0.82 (0.48, 1.38)	1.09 (0.88, 1.34)	0.97 (0.75, 1.25)	0.67 (0.27, 1.65)	0.93 (0.72, 1.21)	1.07 (0.62, 1.85)	1.00 (0.52, 1.88)
High	1.10 (0.88, 1.38)	0.98 (0.78, 1.22)	1.25 (0.78, 2.03)	1.16 (0.94, 1.44)	0.91 (0.71, 1.18)	0.65 (0.27, 1.62)	1.19 (0.92, 1.53)	1.27 (0.76, 2.15)	1.40 (0.78, 2.54)
Very high	1.32 (1.05, 1.70)	0.92 (0.73, 1.15)	1.32 (0.82, 2.13)	1.06 (0.86, 1.32)	0.80 (0.62, 1.04)	0.95 (0.41, 2.17)	1.15 (0.90, 1.49)	1.13 (0.66, 1.94)	1.13 (0.61, 2.09)
P-trend	0.023	0.837	0.098	0.472	<.001	0.871	0.101	0.526	0.478
Cumulative exposure level (µg/m³×hr)[c] (n=2,708)									
Low	1 (Reference)	1 (Reference)	1 (Reference)	1 (Reference)	1 (Reference)	1 (Reference)	1 (Reference)	1 (Reference)	1 (Reference)
Medium	0.91 (0.72, 1.15)	0.89 (0.70, 1.13)	1.48 (0.86, 2.55)	1.50 (1.20, 1.86)	1.15 (0.89, 1.50)	0.71 (0.22, 2.25)	1.07 (0.81, 1.41)	1.09 (0.61, 1.94)	0.96 (0.48, 1.90)
High	1.10 (0.88, 1.39)	1.04 (0.82, 1.31)	1.42 (0.82, 2.46)	1.18 (0.95, 1.46)	1.16 (0.89, 1.51)	1.27 (0.47, 3.44)	1.50 (1.16, 1.95)	1.33 (0.76, 2.32)	1.31 (0.69, 2.49)
Very high	1.30 (1.03, 1.63)	1.30 (1.03, 1.63)	1.98 (1.18, 3.32)	1.59 (1.27, 1.98)	1.10 (0.84, 1.45)	2.50 (1.02, 6.10)	1.55 (1.19, 2.01)	1.41 (0.82, 2.43)	1.54 (0.83, 2.85)
P-trend	0.009	0.013	0.014	0.001	0.473	0.014	<.001	0.160	0.095

Note: Models were adjusted for covariates in Table 2 and additionally adjusted for house mold.
Brain disease: brain and cerebrovascular disease.
[a] Classified by quartile cut-points: 2688, 6468, and 14112 hr.
[b] Classified by quartile cut-points: 293.04, 493.37, and 866.39 µg/m³.
[c] Classified by quartile cut-points: 864130, 2988271, and 8729147 µg/m³×hr.

Table S14. ORs (95% CIs) of reported health conditions by humidifier disinfectant exposures after further adjustment for neighborhood factory or incineration.

Exposures	Pneumonia	Asthma	Cardiovascular disease	Respiratory disease	Otorhinolaryng-ologic disease	Brain disease	Dermatological disease	All cancers	Lung cancer
Chemical type (n=3,958)									
PHMG	1 (Reference)	1 (Reference)	1 (Reference)	1 (Reference)	1 (Reference)	1 (Reference)	1 (Reference)	1 (Reference)	1 (Reference)
PGH	0.80 (0.42, 1.52)	0.87 (0.45, 1.68)	1.16 (0.27, 4.89)	0.76 (0.42, 1.39)	1.99 (1.04, 3.81)	1.73 (0.23, 13.0)	0.88 (0.43, 1.82)	N/A	N/A
CMIT/MIT	0.88 (0.74, 1.04)	1.29 (1.09, 1.53)	1.08 (0.74, 1.56)	1.16 (0.99, 1.37)	1.22 (1.01, 1.47)	1.24 (0.67, 2.27)	0.94 (0.77, 1.14)	0.79 (0.51, 1.21)	0.88 (0.74, 1.04)
Others	0.86 (0.57, 1.29)	1.59 (1.09, 2.33)	0.94 (0.38, 2.36)	1.02 (0.70, 1.49)	1.08 (0.70, 1.68)	2.91 (1.12, 7.54)	0.79 (0.50, 1.26)	0.87 (0.31, 2.45)	0.86 (0.57, 1.29)
Exposure direction (n=4,145)									
Toward the other sides	1 (Reference)	1 (Reference)	1 (Reference)	1 (Reference)	1 (Reference)	1 (Reference)	1 (Reference)	1 (Reference)	1 (Reference)
Toward the face	1.07 (0.93, 1.25)	1.03 (0.89, 1.20)	1.04 (0.75, 1.44)	1.05 (0.91, 1.20)	1.00 (0.84, 1.19)	1.51 (0.83, 2.72)	1.07 (0.90, 1.26)	0.92 (0.66, 1.29)	0.98 (0.67, 1.43)
Exposure proximity (meter) (n=4,142)									
≥ 1m	1 (Reference)	1 (Reference)	1 (Reference)	1 (Reference)	1 (Reference)	1 (Reference)	1 (Reference)	1 (Reference)	1 (Reference)
0.5 to < 1m	0.90 (0.77, 1.06)	0.97 (0.83, 1.15)	1.33 (0.91, 1.95)	1.18 (1.01, 1.38)	1.08 (0.89, 1.31)	1.11 (0.61, 2.03)	1.13 (0.94, 1.37)	1.62 (1.07, 2.43)	1.71 (1.08, 2.71)
< 0.5m	1.06 (0.89, 1.26)	0.91 (0.76, 1.08)	1.40 (0.95, 2.07)	1.01 (0.86, 1.18)	0.99 (0.81, 1.22)	1.08 (0.57, 2.04)	0.90 (0.73, 1.10)	1.57 (1.04, 2.38)	1.65 (1.04, 2.63)
P-trend	0.464	0.268	0.105	0.935	0.888	0.825	0.244	0.053	0.054
Exposure duration (month) (n=3,981)									
< 6 months	1 (Reference)	1 (Reference)	1 (Reference)	1 (Reference)	1 (Reference)	1 (Reference)	1 (Reference)	1 (Reference)	1 (Reference)
6 to < 12 months	1.18 (0.89, 1,55)	1.54 (1.14, 2.08)	0.94 (0.48, 1.85)	1.20 (0.93, 1.55)	1.52 (1.08, 2.14)	1.13 (0.33, 3.88)	1.29 (0.93, 1.79)	0.73 (0.33, 1.61)	1.00 (0.35, 2.86)
12 to < 24 months	1.04 (0.80, 1.34)	1.81 (1.37, 2.39)	1.26 (0.69, 2.28)	1.30 (1.03, 1.65)	1.54 (1.11, 2.12)	1.52 (0.50, 4.62)	1.26 (0.93, 1.72)	1.48 (0.76, 2.87)	2.50 (1.03, 6.06)
≥ 24 months	1.24 (0.97,1.57)	2.01 (1.54, 2.63)	1.35 (0.77, 2.35)	1.46 (1.17, 1.83)	1.90 (1.40, 2.58)	1.97 (0.69, 5.58)	1.57 (1.17, 2.10)	1.52 (0.82, 2.81)	2.73 (1.17, 6.36)
P-trend	0.095	<.001	0.112	<.001	<.001	0.078	0.001	0.021	<.001
Cumulative exposure time (hr)[a] (n=3,896)									
Low	1 (Reference)	1 (Reference)	1 (Reference)	1 (Reference)	1 (Reference)	1 (Reference)	1 (Reference)	1 (Reference)	1 (Reference)
Medium	0.97 (0.80, 1.18)	1.17 (0.96, 1.43)	1.11 (0.93, 1.54)	1.11 (0.93, 1.33)	1.37 (1.09, 1.72)	0.99 (0.43, 2.31)	1.22 (0.98, 1.53)	1.26 (0.79, 2.06)	1.39 (0.79, 2.44)
High	1.07 (0.88, 1.30)	1.30 (1.07, 1.59)	1.31 (0.84, 2.03)	1.24 (1.03, 1.48)	1.20 (0.95, 1.52)	1.54 (0.71, 3.30)	1.07 (0.85, 1.34)	1.48 (0.93, 2.36)	1.85 (1.08, 3.16)
Very high	1.25 (1.03, 1.52)	1.40 (1.14, 1.71)	1.83 (1.21, 2.76)	1.51 (1.26, 1.82)	1.44 (1.14, 1.82)	2.17 (1.05, 4.46)	1.54 (1.23, 1.93)	1.63 (1.04, 2.56)	2.03 (1.21, 3.42)
P-trend	0.015	0.001	0.001	<.001	0.012	0.014	0.001	0.027	0.004
Indoor air concentration ($\mu g/m^3$)[b] (n=2,845)									
Low	1 (Reference)	1 (Reference)	1 (Reference)	1 (Reference)	1 (Reference)	1 (Reference)	1 (Reference)	1 (Reference)	1 (Reference)
Medium	1.10 (0.88, 1.38)	0.81 (0.65, 1.02)	0.82 (0.48, 1.38)	1.09 (0.88, 1.34)	0.97 (0.75, 1.25)	0.67 (0.27, 1.65)	0.93 (0.72, 1.21)	1.07 (0.62, 1.85)	1.00 (0.52, 1.88)
High	1.10 (0.88, 1.38)	0.98 (0.78, 1.22)	1.25 (0.78, 2.03)	1.16 (0.94, 1.44)	0.91 (0.71, 1.18)	0.65 (0.27, 1.62)	1.19 (0.92, 1.53)	1.27 (0.76, 2.15)	1.40 (0.78, 2.54)
Very high	1.32 (1.05, 1.70)	0.92 (0.73, 1.15)	1.32 (0.82, 2.13)	1.06 (0.86, 1.32)	0.80 (0.62, 1.04)	0.95 (0.41, 2.17)	1.15 (0.90, 1.49)	1.13 (0.66, 1.94)	1.13 (0.61, 2.09)
P-trend	0.023	0.837	0.098	0.472	<.001	0.871	0.101	0.526	0.478
Cumulative exposure level ($\mu g/m^3 \times hr$)[c] (n=2,708)									
Low	1 (Reference)	1 (Reference)	1 (Reference)	1 (Reference)	1 (Reference)	1 (Reference)	1 (Reference)	1 (Reference)	1 (Reference)
Medium	0.91 (0.72, 1.15)	0.89 (0.70, 1.13)	1.48 (0.86, 2.55)	1.50 (1.20, 1.86)	1.15 (0.89, 1.50)	0.71 (0.22, 2.25)	1.07 (0.81, 1.41)	1.09 (0.61, 1.94)	0.96 (0.48, 1.90)
High	1.10 (0.88, 1.39)	1.04 (0.82, 1.31)	1.42 (0.82, 2.46)	1.18 (0.95, 1.46)	1.16 (0.89, 1.51)	1.27 (0.47, 3.44)	1.50 (1.16, 1.95)	1.33 (0.76, 2.32)	1.31 (0.69, 2.49)
Very high	1.30 (1.03, 1.63)	1.30 (1.03, 1.63)	1.98 (1.18, 3.32)	1.59 (1.27, 1.98)	1.10 (0.84, 1.45)	2.50 (1.02, 6.10)	1.55 (1.19, 2.01)	1.41 (0.82, 2.43)	1.54 (0.83, 2.85)
P-trend	0.009	0.013	0.014	0.001	0.473	0.014	<.001	0.160	0.095

Note: Models were adjusted for covariates in Table 2 and additionally adjusted for neighborhood factory or incineration.
Brain disease: brain and cerebrovascular disease.
[a] Classified by quartile cut-points: 2688, 6468, and 14112 hr.
[b] Classified by quartile cut-points: 293.04, 493.37, and 866.39 $\mu g/m^3$.
[c] Classified by quartile cut-points: 864130, 2988271, and 8729147 $\mu g/m^3 \times hr$.

Table S15. ORs (95% Cis) of reported health conditions by humidifier disinfectant exposures after further adjustment for occupational exposure.

Exposures	Pneumonia	Asthma	Cardiovascular disease	Respiratory disease	Otorhinolaryng-ologic disease	Brain disease	Dermatological disease	All cancers	Lung cancer
Chemical type (n=3,958)									
PHMG	1 (Reference)	1 (Reference)	1 (Reference)	1 (Reference)	1 (Reference)	1 (Reference)	1 (Reference)	1 (Reference)	1 (Reference)
PGH	0.79 (0.41, 1.50)	0.87 (0.45, 1.69)	1.18 (0.28, 4.98)	0.76 (0.42, 1.39)	1.99 (1.04, 3.81)	1.73 (0.23, 13.0)	0.88 (0.43, 1.82)	N/A	N/A
CMIT/MIT	0.87 (0.74, 1.04)	1.29 (1.09, 1.53)	1.08 (0.74, 1.56)	1.16 (0.99, 1.37)	1.22 (1.01, 1.47)	1.24 (0.67, 2.27)	0.94 (0.77, 1.14)	0.78 (0.51, 1.21)	0.87 (0.74, 1.04)
Others	0.86 (0.57, 1.28)	1.59 (1.09, 2.34)	0.95 (0.38, 2.37)	1.03 (0.70, 1.49)	1.08 (0.70, 1.68)	2.91 (1.12, 7.54)	0.79 (0.50, 1.26)	0.84 (0.30, 2.36)	0.86 (0.57, 1.28)
Exposure direction (n=4,145)									
Toward the other sides	1 (Reference)	1 (Reference)	1 (Reference)	1 (Reference)	1 (Reference)	1 (Reference)	1 (Reference)	1 (Reference)	1 (Reference)
Toward the face	1.08 (0.93, 1.25)	1.03 (0.89, 1.15)	1.04 (0.75, 1.45)	1.05 (0.91, 1.20)	1.00 (0.84, 1.19)	1.51 (0.83, 2.72)	1.07 (0.90, 1.26)	0.93 (0.66, 1.30)	0.99 (0.68, 1.44)
Exposure proximity (meter) (n=4,142)									
≥ 1m	1 (Reference)	1 (Reference)	1 (Reference)	1 (Reference)	1 (Reference)	1 (Reference)	1 (Reference)	1 (Reference)	1 (Reference)
0.5 to < 1m	0.90 (0.76, 1.06)	0.97 (0.83, 1.15)	1.34 (0.91, 1.96)	1.18 (1.01, 1.38)	1.08 (0.89, 1.31)	1.12 (0.61, 2.06)	1.13 (0.94, 1.37)	1.63 (1.08, 2.45)	1.73 (1.10, 2.74)
< 0.5m	1.05 (0.89, 1.25)	0.91 (0.76, 1.08)	1.41 (0.95, 2.08)	1.01 (0.86, 1.18)	0.99 (0.81, 1.22)	1.10 (0.58, 2.08)	0.90 (0.73, 1.10)	1.58 (1.04, 2.40)	1.67 (1.05, 2.66)
P-trend	0.478	0.272	0.098	0.928	0.906	0.784	0.251	0.049	0.050
Exposure duration (month)(n=3,981)									
< 6 months	1 (Reference)	1 (Reference)	1 (Reference)	1 (Reference)	1 (Reference)	1 (Reference)	1 (Reference)	1 (Reference)	1 (Reference)
6 to < 12 months	1.17 (0.89, 1,54)	1.54 (1.14, 2.08)	0.95 (0.48, 1.86)	1.20 (0.93, 1.55)	1.52 (1.08, 2.15)	1.14 (0.33, 3.92)	1.29 (0.93, 1.79)	0.73 (0.33, 1.61)	1.00 (0.35, 2.87)
12 to < 24 months	1.04 (0.80, 1.34)	1.81 (1.37, 2.39)	1.25 (0.69, 2.27)	1.30 (1.03, 1.65)	1.53 (1.11, 2.11)	1.52 (0.50, 4.63)	1.26 (0.93, 1.72)	1.49 (0.76, 2.87)	2.50 (1.03, 6.06)
≥ 24 months	1.24 (0.98,1.58)	2.01 (1.54, 2.62)	1.35 (0.78, 2.36)	1.46 (1.17, 1.82)	1.90 (1.40, 2.58)	2.01 (0.71, 5.71)	1.58 (1.18, 2.11)	1.52 (0.82, 2.83)	2.75 (1.18, 6.39)
P-trend	0.081	<.001	0.108	<.001	<.001	0.069	<.001	0.019	<.001
Cumulative exposure time (hr)[a] (n=3,896)									
Low	1 (Reference)	1 (Reference)	1 (Reference)	1 (Reference)	1 (Reference)	1 (Reference)	1 (Reference)	1 (Reference)	1 (Reference)
Medium	0.97 (0.80, 1.18)	1.17 (0.96, 1.43)	1.11 (0.93, 1.54)	1.11 (0.93, 1.33)	1.37 (1.09, 1.72)	0.99 (0.43, 2.31)	1.22 (0.98, 1.53)	1.26 (0.79, 2.06)	1.39 (0.79, 2.44)
High	1.07 (0.88, 1.30)	1.30 (1.07, 1.59)	1.31 (0.84, 2.03)	1.24 (1.03, 1.48)	1.20 (0.95, 1.52)	1.54 (0.71, 3.30)	1.07 (0.85, 1.34)	1.48 (0.93, 2.36)	1.85 (1.08, 3.16)
Very high	1.25 (1.03, 1.52)	1.40 (1.14, 1.71)	1.83 (1.21, 2.76)	1.51 (1.26, 1.82)	1.44 (1.14, 1.82)	2.17 (1.05, 4.46)	1.54 (1.23, 1.93)	1.63 (1.04, 2.56)	2.03 (1.21, 3.42)
P-trend	0.015	0.001	0.001	<.001	0.012	0.014	0.001	0.027	0.004
Indoor air concentration (μg/m^3)[b] (n=2,845)									
Low	1 (Reference)	1 (Reference)	1 (Reference)	1 (Reference)	1 (Reference)	1 (Reference)	1 (Reference)	1 (Reference)	1 (Reference)
Medium	1.10 (0.88, 1.38)	0.81 (0.65, 1.02)	0.82 (0.48, 1.38)	1.09 (0.88, 1.34)	0.97 (0.75, 1.25)	0.67 (0.27, 1.65)	0.93 (0.72, 1.21)	1.07 (0.62, 1.85)	1.00 (0.52, 1.88)
High	1.10 (0.88, 1.38)	0.98 (0.78, 1.22)	1.25 (0.78, 2.03)	1.16 (0.94, 1.44)	0.91 (0.71, 1.18)	0.65 (0.27, 1.62)	1.19 (0.92, 1.53)	1.27 (0.76, 2.15)	1.40 (0.78, 2.54)
Very high	1.32 (1.05, 1.70)	0.92 (0.73, 1.15)	1.32 (0.82, 2.13)	1.06 (0.86, 1.32)	0.80 (0.62, 1.04)	0.95 (0.41, 2.17)	1.15 (0.90, 1.49)	1.13 (0.66, 1.94)	1.13 (0.61, 2.09)
P-trend	0.023	0.837	0.098	0.472	<.001	0.871	0.101	0.526	0.478
Cumulative exposure level (μg/m^3×hr)[c](n=2,708)									
Low	1 (Reference)	1 (Reference)	1 (Reference)	1 (Reference)	1 (Reference)	1 (Reference)	1 (Reference)	1 (Reference)	1 (Reference)
Medium	0.91 (0.72, 1.15)	0.89 (0.70, 1.13)	1.48 (0.86, 2.55)	1.50 (1.20, 1.86)	1.15 (0.89, 1.50)	0.71 (0.22, 2.25)	1.07 (0.81, 1.41)	1.09 (0.61, 1.94)	0.96 (0.48, 1.90)
High	1.10 (0.88, 1.39)	1.04 (0.82, 1.31)	1.42 (0.82, 2.46)	1.18 (0.95, 1.46)	1.16 (0.89, 1.51)	1.27 (0.47, 3.44)	1.50 (1.16, 1.95)	1.33 (0.76, 2.32)	1.31 (0.69, 2.49)
Very high	1.30 (1.03, 1.63)	1.30 (1.03, 1.63)	1.98 (1.18, 3.32)	1.59 (1.27, 1.98)	1.10 (0.84, 1.45)	2.50 (1.02, 6.10)	1.55 (1.19, 2.01)	1.41 (0.82, 2.43)	1.54 (0.83, 2.85)
P-trend	0.009	0.013	0.014	0.001	0.473	0.014	<.001	0.160	0.095

Note: Models were adjusted for covariates in Table 2 and additionally adjusted for occupational exposure.

Brain disease: brain and cerebrovascular disease.

[a] Classified by quartile cut-points: 2688, 6468, and 14112 hr.

[b] Classified by quartile cut-points: 293.04, 493.37, and 866.39 μg/m^3.

[c] Classified by quartile cut-points: 864130, 2988271, and 8729147 μg/m^3×hr.

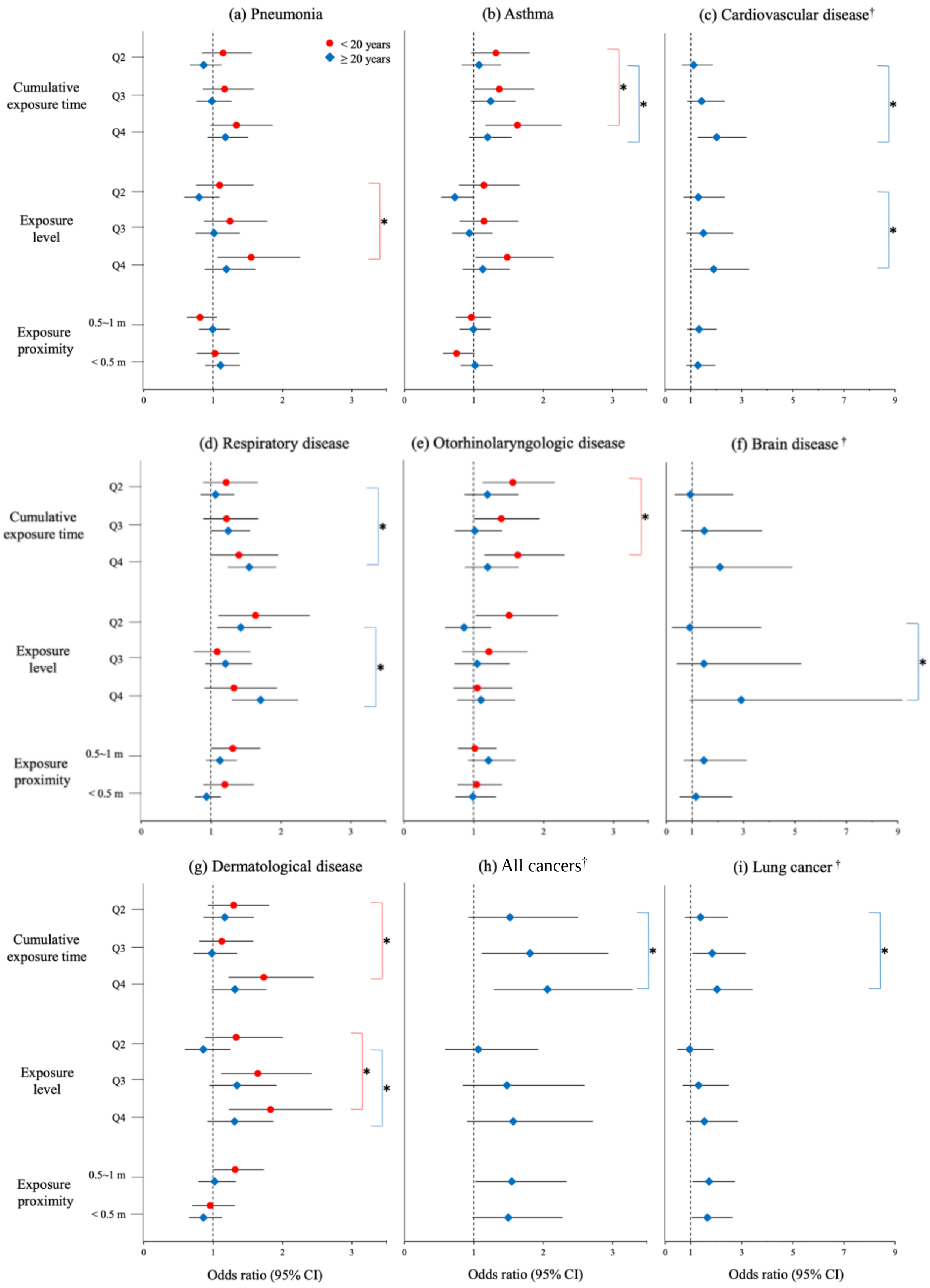

Figure S1. ORs (95% CIs) of various diseases by humidifier disinfectant exposures in the age group of < 20 and ≥ 20 years.
Brain disease: brain and cerebrovascular disease. Q, quartile. Cut-points: 2688, 6468, and 14112 hr for cumulative exposure time; 864130, 2988271, and 8729147 μg/m³×hr for cumulative exposure level.
Reference: Q1 for cumulative exposure time and cumulative exposure level; ≥ 1 m for exposure proximity.
*Statistical significance (p for trend < 0.05).
†Analysis for the age group of < 20 years was unavailable.

Supplementary Materials, REFERENCE.

1. Seong HU, Cho SD, Park SY, et al. Nationwide survey on the prevalence of allergic diseases according to region and age. *Pediatric Allergy and Respiratory Disease* 2012; **22**(3): 224-31.
2. Koh TH, Park J-T, Kwon D, Kwak K. Association between use of humidifier disinfectant and allergic rhinitis in Korean children: a cross-sectional study based on the eighth Panel Study on Korean Children (PSKC). *Annals of Occupational and Environmental Medicine* 2020; **32**(1).
3. Ha J, Lee SW, Yon DK. Ten-Year trends and prevalence of asthma, allergic rhinitis, and atopic dermatitis among the Korean population, 2008–2017. *Clinical and experimental pediatrics* 2020; **63**(7): 278.
